# Frequency of pleural effusion in dengue patients by severity, age and imaging modality: a systematic review and meta-analysis

**DOI:** 10.1186/s12879-023-08311-y

**Published:** 2023-05-15

**Authors:** Molly D. Kaagaard, Luan Oliveira Matos, Marliton V. P. Evangelista, Alma Wegener, Anna Engell Holm, Lasse S. Vestergaard, Suiane C. N. Do Valle, Odilson M. Silvestre, Marcus Vinícius Guimarães Lacerda, Rodrigo Medeiros de Souza, Flavia Barreto dos Santos, Tor Biering-Sørensen, Philip Brainin

**Affiliations:** 1 Multidisciplinary Center, Federal University of Acre, Campus Floresta, Cruzeiro do Sul, Acre, Brazil; 2 Cardiovascular Non-Invasive Imaging Research Laboratory, Department of Cardiology, Herlev-Gentofte University Hospital, Hellerup, Denmark; 3 Department of Bacteria, Parasites and Fungi, National Malaria Reference Laboratory, Statens Serum Institut, Copenhagen, Denmark; 4 Health and Sport Science Center, Federal University of Acre, Rio Branco, Acre, Brazil; 5 Foundation of Tropical Medicine Dr Heitor Vieira Dourado, Fiocruz, Manaus Brazil; 6 University of Texas Medical Branch, Galveston, USA; 7 Laboratory of Host-Virus Interactions, Oswaldo Cruz Institute, Fiocruz, Rio de Janeiro, RJ Brazil; 8 Faculty of Biomedical Sciences, Copenhagen University, Copenhagen, Denmark; 9 Sound Bioventures, Hellerup, Denmark

**Keywords:** Dengue fever, Pleural effusion, Ultrasound, Plasma leakage

## Abstract

**Background:**

Identification of pleural effusion (PE) in dengue infection is an objective measure of plasma leakage and may predict disease progression. However, no studies have systematically assessed the frequency of PE in patients with dengue, and whether this differs across age and imaging modality.

**Methods:**

We searched Pubmed, Embase Web of Science and Lilacs (period 1900–2021) for studies reporting on PE in dengue patients (hospitalized and outpatient). We defined PE as fluid in the thoracic cavity detected by any imaging test. The study was registered in PROSPERO (CRD42021228862). Complicated dengue was defined as hemorrhagic fever, dengue shock syndrome or severe dengue.

**Results:**

The search identified 2,157 studies of which 85 studies were eligible for inclusion. The studies (n = 31 children, n = 10 adults, n = 44 mixed age) involved 12,800 patients (30% complicated dengue). The overall frequency of PE was 33% [95%CI: 29 to 37%] and the rate of PE increased significantly with disease severity (P = 0.001) such that in complicated vs. uncomplicated dengue the frequencies were 48% and 17% (P < 0.001). When assessing all studies, PE occurred significantly more often in children compared to adults (43% vs. 13%, P = 0.002) and lung ultrasound more frequently detected PE than conventional chest X-ray (P = 0.023).

**Conclusions:**

We found that 1/3 of dengue patients presented with PE and the frequency increased with severity and younger age. Importantly, lung ultrasound demonstrated the highest rate of detection. Our findings suggest that PE is a relatively common finding in dengue and that bedside imaging tools, such as lung ultrasound, potentially may enhance detection.

**Supplementary Information:**

The online version contains supplementary material available at 10.1186/s12879-023-08311-y.

## Background

Dengue is a viral, vector-borne disease present in over 100 countries in the tropics and subtropics, affecting more than 105 million people each year [1]. The clinical presentation ranges from asymptomatic disease, over mild infection with fever and joint pain, to severe dengue with plasma leakage and bleeding, potentially leading to shock and death. In 1997 the World Health Organization (WHO) categorized the clinical presentation of dengue infection as either dengue fever, dengue hemorrhagic fever or dengue shock syndrome [2]. This was changed in 2009 to three novel categories: dengue without warning signs, dengue with warning signs and severe dengue [3]. While the 1997 classification recognized plasma leakage as a key parameter of dengue severity, the 2009 definition emphasized individual organ dysfunction as more important, and only plasma leakage leading to shock or respiratory distress is classified as a severe case [4]. Noteworthy, many researchers and clinicians continue to use the old classification [5], arguing that it is more reliable, and that delayed identification of plasma leakage is a common cause of dengue mortality [6]. Indeed, a recent meta-analysis showed that clinical signs of plasma leakage was a strong predictor of progression to severe dengue and that timely identification may lead to improved management [7].

Pleural effusion (PE) is a common manifestation of plasma leakage in dengue [8] and is diagnosed by imaging diagnostic tests. As of today, only small-scale studies have reported sporadically on PE in dengue which has been in the range of 2–100% [9, 10]. However, no study has systematically evaluated the frequency of PE in dengue and therefore it remains unknown to what extent clinicians can use PE as a marker of disease severity. The aim of this study was to determine the frequency of PE in dengue across disease severity, age groups and evaluate whether certain imaging diagnostic tests would enhance detection of PE.

## Methods

We included full-text clinical studies according to the Preferred Reporting Items for Systematic Reviews and Meta-Analyses (PRISMA) [11]. The study was registered in PROSPERO (registration No.: CRD42021228862) [12].

### Criteria

We assessed studies of children and adults with a positive test for dengue determined by rapid diagnostic tests and Enzyme-Linked Immunosorbent Assay (ELISA) showing presence of NS1 antigen, IgM or IgG, reverse transcription polymerase chain reaction, or hemagglutination inhibition. Studies were included if they reported PE assessed by a paraclinical imaging diagnostic: chest X-ray (CXR), ultrasound or computed tomography (CT). Exclusion criteria were the following: studies in other languages than English, French, Spanish, or Portuguese, known concomitant infection (such as malaria, COVID-19 or other pulmonary infections), known pulmonary disease or heart failure at baseline, known heart/lung/kidney transplant, recent chest trauma or surgery (< 4 weeks ago) or catheter attached to the thorax.

### Search and review

We searched PubMed, Embase, Web of Science and Lilacs from 1900 to 2021 using a broad search string including “Dengue virus” OR “Dengue infection” OR “DENV” AND “Pleural effusion” OR “Pulmonary edema” AND “Diagnostic imaging” OR “Ultrasound”. The full search string is presented in Supplemental Table 1. Furthermore, we examined bibliographies of included and excluded studies. Two independent reviewers (MDK, MVPE**)** performed the literature search and screened titles and abstracts to identify potentially eligible articles. The full-text articles of these were screened by MDK and PB, and finally included or excluded. Finally, decisions of the reviewers were compared, and disagreement was resolved by consensus.

### Data collection

MDK and PB extracted data from the included articles, which involved country, year, sample size, baseline characteristics (sex, age, inpatient/outpatient), diagnostic test, clinical complications, serotypes, disease severity and mortality. Children were defined as ≤ 17 years old and disease severity was assessed using the classification provided in the study (either WHO’s 1997 or 2009 algorithm). For the purpose of this study, we stratified the population into two severity groups: complicated and uncomplicated dengue. We defined complicated dengue as dengue hemorrhagic fever or dengue shock syndrome (1997 classification) and severe dengue (2009 classification) (Supplemental Fig. 1). All other cases of dengue were classified as uncomplicated. For studies that included patients with mixed dengue severity, we sought to extract individual patient data when possible. Moreover, we extracted information about the imaging methods for detection of PE. If studies assessed PE several times during hospitalization, we used the imaging test performed closest to defervescence where the risk of plasma leakage is considered highest [13].

### Bias assessment

Quality of included studies was assessed by MDK and PB, using the NIHLBI tool [14] for observational cohort and cross-sectional studies (Supplemental Table 2).

### Statistics

Frequencies of PE were extracted from individual studies and afterwards we applied a random-effects model to assess the pooled frequency of PE across studies. We used the *metaprop* command in STATA [15] and assessed pooled estimates in four study categories: (i) Across all studies, (ii) by age group (children vs. adults), (iii) by severity (complicated vs. uncomplicated) and (iv) by imaging diagnostic test. Heterogeneity was assessed using the I^2^ value and forest plots were constructed to display frequency estimates. P-values for heterogeneity were considered significant when below 0.05. Frequencies of PE across subgroups, such as CXR vs. ultrasound vs. CT, were compared using Cuzick’s non-parametric test for trend [16], whereas individual subgroups were compared using Wilcoxon rank sum test. Meta regression models were conducted to investigate whether geographical region and classification algorithm influenced the frequency of PE. We considered P-values < 0.05 as significant. Statistical analyses were performed with STATA (version 13.1, College Station, TX).

## Results

**Fig. 1:**
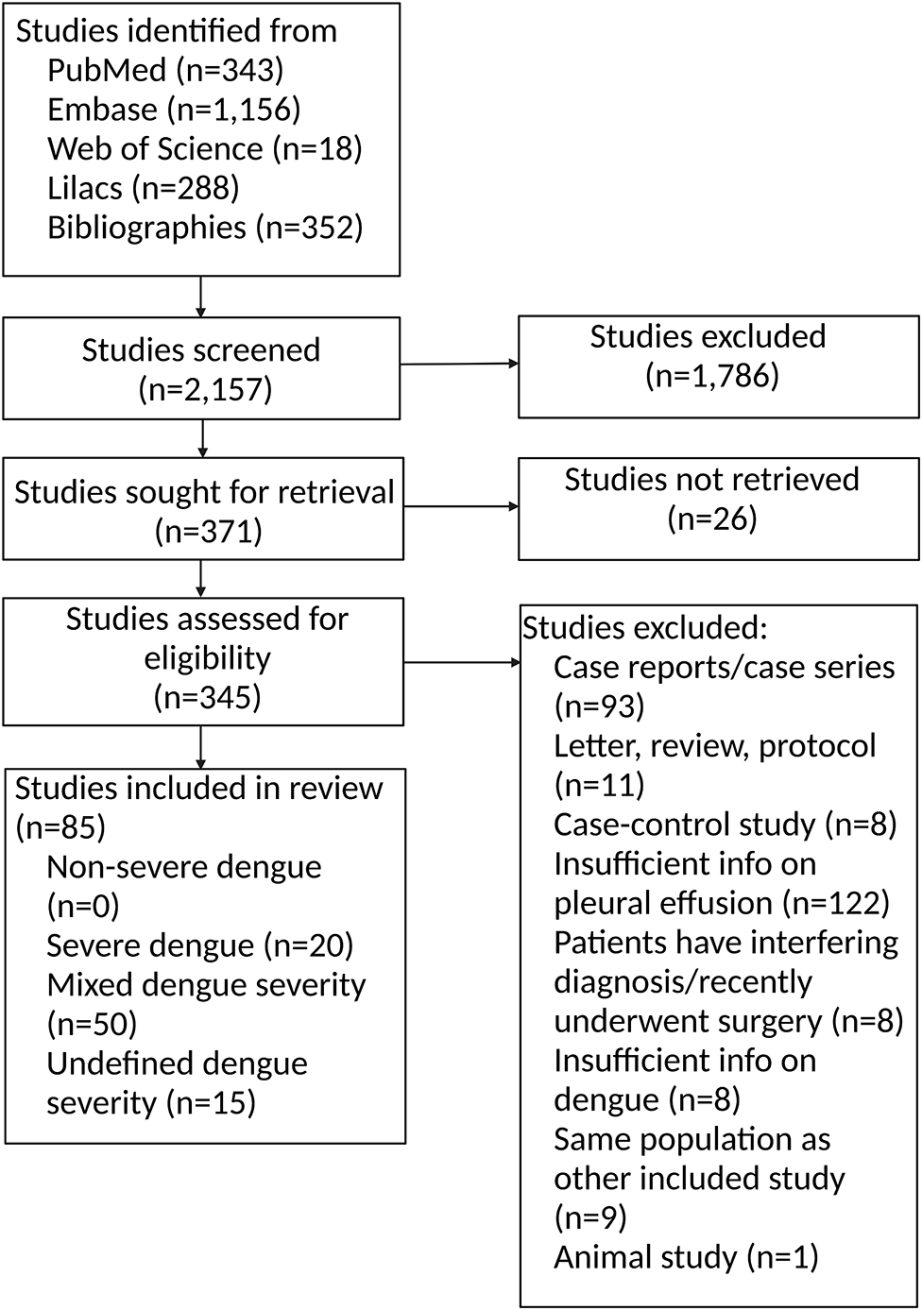
Flowchart of inclusion and exclusion Overview of search results and reasons for exclusion

The literature search and bibliography screening yielded 2,157 studies. Of these, 1,786 (82%) were excluded on the basis of title and abstract, and 26 studies could not be retrieved in full text (Fig. 1). We assessed 345 studies in full text and 85 were included (published from 1991 to 2020; Supplemental Table 3) [4, 9, 10, 17–98].

We found an overall acceptable degree of bias among the included studies, where 49% were rated ‘good’ using the applied tool, and 42% were rated ‘fair’.

The studies more often assessed dengue severity using the 1997 WHO classification (n = 48) than the 2009 classification (n = 20), and 17 studies did not specify the classification. Three studies were published before 1997 [47, 50, 69]. The studies included 12,800 patients (49% male, mean age 24 years) and a majority had mixed adults and children (n = 34), followed by studies of children (n = 31), adults (n = 10) and unspecified age (n = 10). Most included hospitalized patients (n = 66), of which four were conducted in intensive care units, three were from emergency departments and eight had a mix of hospitalized and ambulatory patients. Geographically, a majority of studies were conducted in South-East Asia (n = 68) (Supplemental Fig. 2) and most used ELISA for IgM/IgG seroconversion to diagnose dengue (n = 47) (Supplemental Table 3). Few studies reported on dengue and associated clinical complications (Supplemental Table 4) and few (n = 13) had data on serotype (Supplemental Table 5) for which there was no clear pattern associated with PE.

### Pooled frequency of pleural effusion

**Fig. 2:**
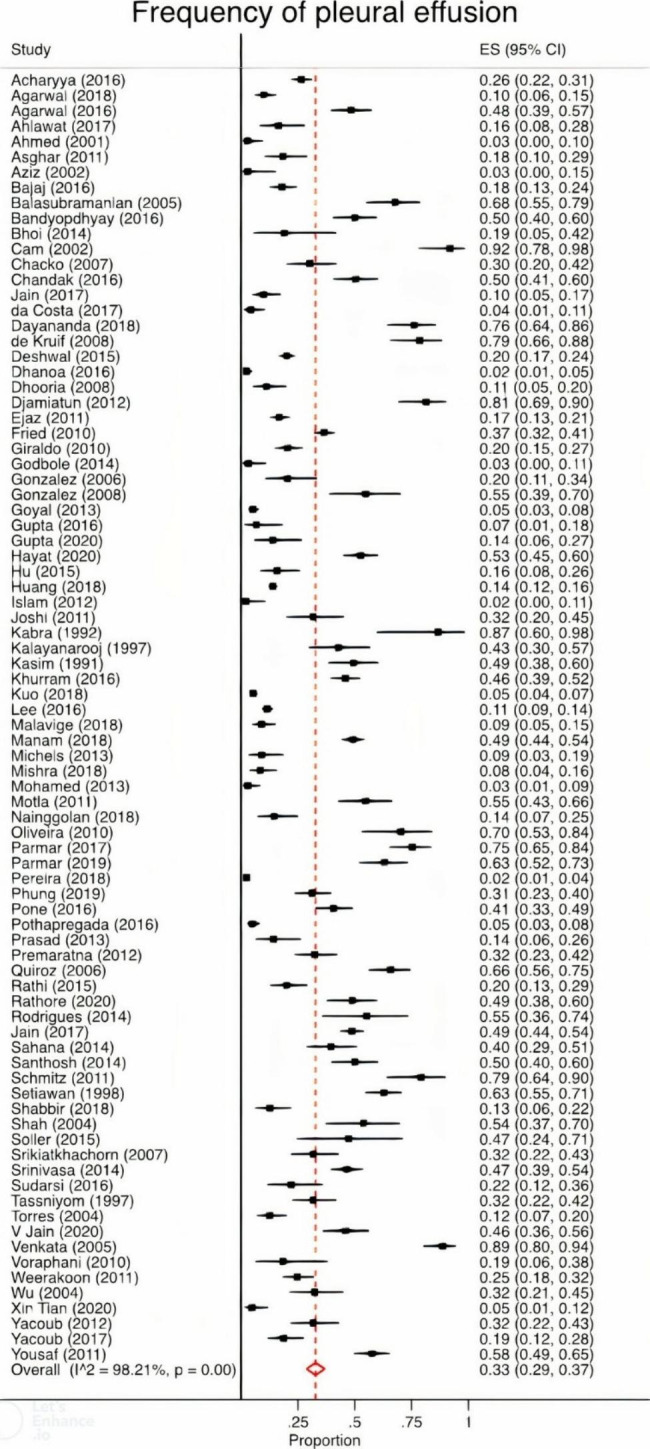
Pooled frequency of PE Meta-analysis of PE based on all included studies

The meta-analysis of all studies yielded a pooled PE frequency of 33% [95%CI 29 to 37%] (Fig. 2) and heterogeneity was considered high with an I^2^ of 98%, P < 0.01. Among three studies assessing mortality, survivors had lower frequency of PE (14% [95% CI -24 to 52%]) than those who died (46% [95%CI -13 to 104%]). A meta-regression showed that the geographical region in which studies were conducted did not affect occurrence of PE (P = 0.32). By contrast, classification was associated with PE such that the highest frequency occurred in studies using the 1997 classification (P = 0.004).

### Pleural effusion by severity

Uncomplicated: Studies of uncomplicated dengue (n = 26) had the lowest number of patients (n = 2,431 patients, age range 0–93 years), and they displayed the lowest pooled frequency of PE (17% [95%CI 12 to 22%]). Mixed: Studies of mixed dengue severity (n = 28) had more patients (n = 5,764 patients; age range 0–93 years, 61% male,) and the frequency of PE was lower (29% [95%CI 22 to 35%]). Complicated: In studies of complicated dengue (n = 47 studies, n = 2,978 patients, age range 0–92 years, 49% males) the pooled frequency of PE was 48% [95%CI 38 to 59%].

The frequency of PE increased with severity (P trend = 0.001, Table 1) and patients with complicated infection had significantly higher rate of PE compared to uncomplicated dengue (48% vs. 17%, P < 0.001). Heterogeneity in these analyses was high (I^2^ from 96 to 99%, P < 0.01). Studies using the 1997 WHO classification reported higher frequencies of PE (38% [95%CI 32 to 45%]) compared to studies using the 2009 classification (17% [95%CI 14 to 21%]) (P = 0.003), thereby confirming the findings from the metaregression analysis.

### Pleural effusion by age group

The frequency of PE was significantly higher in children compared to adults (43% vs. 13%, P = 0.002, Table 1). Specifically for children, PE occurred significantly more in complicated vs. uncomplicated disease (58% vs. 12%, P < 0.001), while this was not the case among adults (P = 0.44). The analyses displayed high heterogeneity with I^2^ values ranging from 96 to 99%, P < 0.01.

### Pleural effusion by imaging test

Diagnostic tests to identify PE involved ultrasound (n = 44), CXR (n = 37), combined ultrasound and CXR (n = 10) and CT (n = 2). Studies using ultrasound reported a pooled frequency of PE of 38% [95%CI 31 to 46%], CXR studies 28% [95%CI 22 to 34%] and mixed ultrasound/CXR 26% [95%CI 19 to 33%]. Two studies using CT reported the highest frequency of PE: 59% [95%CI 47 to 71%]. Frequency of PE varied significantly across imaging modalities (P trend = 0.010; Table 1), such that CT and ultrasound more often identified PE compared to CXR. Ultrasound studies also detected more PE compared to CXR (P = 0.023) when excluding CT and mixed studies.

**Table 1:** Summary table of PE frequencies by severity, age group and imaging modality

Category	Mean age, years (range)	Children, n(%)	Male, %	Hospitalized, n(%)	Secondary infection, %	Ultrasound, n(%)	Frequency of PE*	Heterogeneity (I^2^)
**Severity**							P-trend <0.001	
Uncomplicated	12 (8 to 36)	13 (50%)	-	25 (96%)	52	11 (42%)	17% [95%CI 12 to 22%]	96%
References: [10, 17, 20, 30, 32, 39, 43, 49, 53, 56, 58, 65, 67, 71, 74–76, 98]								
Mixed	28 (8 to 34)	10 (36%)	59	23 (82%)	55	13 (46%)	29% [95%CI 22 to 35%]	99%
References: [9, 18, 21, 23, 25, 27, 31, 33, 34, 36, 37, 42, 44, 46, 48, 52, 55, 57, 63, 68, 72, 81–83, 85, 89–91]								
Complicated	10 (8 to 32)	23 (49%)	53	44 (94%)	82	18 (38%)	48% [95%CI 38 to 59%]	98%
References: [10, 17, 19, 20, 28–30, 32, 34, 39, 41, 43, 45, 47, 49–51, 53, 56, 58, 62, 64–67, 69–71, 73–77, 79, 80, 84, 86–88, 93, 95–98]								
**Age group**							P-trend = 0.005	
Children	8 (8 to 10)	-	52	42 (89%)	82	19 (40%)	43% [95%CI 34 to 51%]	98%
References: [10, 19, 20, 24, 25, 28, 29, 31, 32, 46–50, 62, 66–69, 71, 74, 75, 77, 80, 82–85, 87, 90, 91, 98]								
Mixed	32 (29 to 37)	-	64	36 (88%)	42	23 (56%)	39% [95%CI 29 to 49%]	99%
References: [9, 17, 18, 22, 27, 30, 33–37, 40, 43, 45, 51, 55, 56, 59, 60, 63, 64, 70, 72, 73, 76, 78, 79, 81, 88, 93, 95, 97]								
Adults	41 (32 to 50)	-	54	11 (85%)	66	8 (62%)	13% [95%CI 9 to 17%]	96%
References: [21, 24, 26, 38, 42, 52–54, 61, 65, 94]								
**Imaging**							P-trend = 0.010	
Ultrasound	28 (10 to 36)	14 (32%)	61	35 (80%)	47	-	38% [95%CI 31 to 46%]	99%
References: [4, 9, 17, 20–22, 24, 34, 35, 37–40, 51, 54–56, 59–65, 68, 69, 72–75, 78, 80, 81, 83–85, 90–92, 94, 95, 97, 98]								
Chest X-ray	8 (8 to 26)	19 (51%)	60	34 (92%)	81	-	28% [95%CI 22 to 34%]	98%
References: [10, 18–20, 22, 23, 26–31, 33, 34, 41, 43, 45–50, 57, 58, 66, 71, 74, 77, 82, 84, 86–89, 93, 96, 98]								
Mix ultrasound and chest X-ray	45 (38 to 50)	3 (30%)	50	8 (80%)	84	-	26% [95%CI 19 to 33%]	98%
References: [25, 32, 36, 42, 44, 52, 53, 67, 70, 79]								
Computed tomography	58 (58 to 58)	0 (0%)	62	2 (100%)	34	-	59% [95%CI 47 to 71%]	-
References: [41, 76]								

*P was calculated using Cuzick’s non-parametric test for trend [16]

CI = confidence interval, PE = pleural effusion

## Discussion

Plasma leakage is associated with mortality in dengue infection [7] and the most common manifestation is PE [2]. Hence, early recognition of PE is useful for risk stratification and may facilitate rapid initiation of treatment. In this meta-analysis we showed that PE occurred in up to one third of all dengue cases, increased with severity and more often appeared in children. Furthermore, we found that ultrasound detected higher rates of PE as compared to CXR.

Present studies of plasma leakage in dengue are of heterogenous nature, observational and contain large differences in their estimates of PE. In two ultrasound studies from India, Venkata et al [90] reported a PE frequency of 5%, whereas Pothapregada et al [68] identified PE in 89%. Although the two studies share similar characteristics (imaging test, population, region), they indicate great variation in the reporting of an important complication to dengue. A recent meta-analysis found that PE and ascites were predictors of progression to severe dengue [7], which is comparable to our findings, where the frequency of PE increased with dengue severity. However, the fact that PE was present in nearly 20% of patients with uncomplicated dengue – and in 33% of all cases – may question its use as a prognostic factor. Severe dengue is characterized by large plasma leakage, whereas a small leakage does not have any clinical impact and may be more common than previously thought [4]. It is possible that the size of the effusion, or earlier detection of PE, could represent a better prognostic factor. This hypothesis was supported by Srikiatkhachorn et al, who demonstrated that the size of PE, assessed by ultrasound, was directly associated with dengue severity (cross-sectional width of PE in uncomplicated = 1 mm and complicated = 24 mm) [84]. Data on this was rarely reported in the assessed studies and could thus not be analyzed, but future studies should delineate whether quantification of PE by ultrasound offers additional clinical and/or prognostic value.

Fried et al [31] hypothesized that children have a greater risk of PE, which is in line with the results in this study (Table 1). A potential reason is that younger age is associated with severe dengue [5], and the risk of hemorrhagic fever increases with 8% for each year decrease in age [7]. Other reasons to account for this difference may be found in the underlying mechanisms of plasma leakage. A proposed mechanism is dysfunction of endothelial glycocalyx [99]. This theory has emerged because leakage often resolves suddenly, making endothelial apoptosis/dysfunction less likely. The free NS1 and virus particles bind to the glycocalyx, which allows leakage of plasma proteins such as albumin. The resulting fall in osmotic pressure pulls plasma to the interstitial space and causes PE [99] (Supplemental Fig. 4). Furthermore, NS1 binds complement and causes release of vasoactive cytokines, increasing vascular permeability [100]. A smaller resistance to plasma leakage in children could be caused by age dependent endothelial differences. Young mammals have a larger microvascular surface per unit volume of skeletal muscle compared to adults, which means that endothelial dysfunction leads to more plasma leakage. Furthermore, microvessels in development have been suggested to be more permeable to plasma [99]. A study by Gamble et al [101] examined vascular permeability in dengue patients and controls at different ages. Vascular permeability was three times higher in healthy children compared to young adults, and the value was approximately 50% higher when infected with dengue. However, no difference in vascular function was observed in those with and without shock, highlighting the need to improve our understanding of the pathophysiology of plasma leakage in children. Higher risk of severe infection and frequency of PE in children may suggest that the initial diagnostic workup in dengue should be age specific, which is in contrast to the current guidelines from WHO [3].

Our findings indicate that ultrasound detects PE more often than CXR (38% vs. 28%). A study by Prina et al [102] found that PE was detected in 93% of cases with ultrasound and only in 47% when using CXR. Moreover, other studies have shown that ultrasound may detect smaller effusions (as low as ~ 20 mL), whereas effusions by CXR are visible when exceeding 200 mL [102]. In the setting of dengue, ultrasound could prove useful as it is faster compared to CXR, handheld devices may be used bedside in emergency departments and even non-physician personnel can acquire and interpret lung ultrasound images after brief training sessions [103]. This could render it particularly useful for risk stratification in areas with few resources. Studies using CT had an even higher frequency of PE, as would be expected since CT has a very high sensitivity for such changes. However, it is also more expensive and not practical to conduct CT scans on every dengue patient during an epidemic, thus potentially making ultrasound a more cost beneficial tool.

Interestingly, the WHO classification algorithm (1997 vs. 2009) was associated with PE such that studies using the 1997 classification reported the highest frequencies of PE. Possible explanations are that diagnostics for PE have become more widespread and used at earlier and milder stages of disease and a tendency towards a more restricted fluid therapy in recent years.

The results from this meta-analysis emphasizes that although PE is a relatively common complication in dengue, it varies according to severity, age and is influenced by imaging modality. So far, classification of dengue severity is based on clinical evidence of fluid accumulation. However, due to the high quality and low cost of ultrasound for detecting PE, it is worthwhile to consider if paraclinical evaluation could allow for a more precise risk assessment. Future studies should aim to determine if paraclinical assessment of PE is superior to that of clinical evaluation and whether it should be assessed on a qualitative vs. quantitative basis (i.e., volume cut-off) and if regional localization matters in dengue. Moreover, it is of importance to elucidate if detection of PE in children offers enhanced opportunity for risk stratification and disease monitoring, considering children’s vulnerability to severe disease. Finally, there is a need to streamline the reporting of PE to reduce heterogeneity in observational studies of dengue.

### Limitations

We only included studies which reported on PE and this may lead to an overestimation of the real frequency. All of our analyses displayed considerable heterogeneity as demonstrated by high I^2^ values, indicating high variability in the reporting of PE. This could be due to differences in study design, included populations, reporting of outcome measures, disease severity and dengue classification algorithm. The high amount of heterogeneity may influence our results and render them less reliable. Furthermore, several included studies lacked information on age, severity and individual reporting of whether ultrasound or CXR had been used. Consequently, we had to include ‘mixed’ categories of age, severity and imaging method. In addition, a majority of studies did not report on mortality and dengue serotype, thus not permitting us to examine if PE was linked to these parameters. PE is known to occur mostly during defervescence, but many studies did not report timing for assessment. The few studies that measured PE at different time points found significant differences between defervescence and the febrile phase (Yousaf et al: 58% vs. 46%, Venkata et al: 72% vs. 6%). We defined our own categories of dengue (complicated vs. uncomplicated) based on the 1997 and 2009 WHO classification instead of using one of the existing categories. This is not clinically optimal, but we deemed it necessary as we did not have access to individual patient data. We found a significantly higher frequency of PE in patients where the 1997 classification was used, compared to those where the 2009 classification was used. An important observation is that the 2009 classification possibly is more sensitive and less specific, leading to more patients classified with severe dengue but without PE.

## Conclusion

To our knowledge, this is the first meta-analysis to assess the frequency of PE in dengue. We found that 33% of all dengue cases displayed PE, and this was significantly higher in children (43%) and in patients with complicated infection (48%). Furthermore, lung ultrasound more often detected PE compared to CXR. Identification of PE by lung ultrasound in dengue may represent a useful tool to assess disease severity and risk stratify patients in remote environments without access to conventional imaging methods.

## Electronic supplementary material

Below is the link to the electronic supplementary material.

Supplementary Material 1

## Data Availability

The dataset used and/or analysed during the current study is available from the corresponding author on reasonable request.

## List of Abbreviations

CT: Computed tomography
CXR: Chest X-ray
ELISA: Enzyme-Linked Immunosorbent Assay
PE: Pleural effusion
