# Supplementary material for: Frequency of pleural effusion in dengue patients by severity, age and imaging modality: a systematic review and meta-analysis
Source: BMC Infect Dis. 2023 May 15;23:327. doi: 10.1186/s12879-023-08311-y (PMC10184094; doi:10.1186/s12879-023-08311-y)
Supplement: Supplementary file 1 — Supplementary Material 1 [file 12879_2023_8311_MOESM1_ESM.docx]

**Supplemental material**

**Frequency of pleural effusion by severity, age and imaging modality in dengue patients: A systematic review and meta-analysis**

Molly D. Kaagaard, Luan Oliveira Matos, Marliton V. P. Evangelista, Alma Wegener, Anna Engell Holm, Lasse S. Vestergaard, Suiane C. N. Do Valle, Odilson M. Silvestre, Marcus Vinícius Guimarães Lacerda, Rodrigo Medeiros de Souza, Flavia Barreto dos Santos, Tor Biering-Sørensen, Philip Brainin

**Content**

[Supplemental Figure 1: Classification of disease severity 2](#_Toc130148037)

[Supplemental Figure 2: Geographical distribution of included studies 3](#_Toc130148038)

[Supplemental Figure 3: Frequency of pleural effusion by age and disease severity 3](#_Toc130148039)

[Supplemental Figure 4: Mechanisms of pleural effusion 4](#_Toc130148040)

[Supplemental Table 1: Search strings for databases 5](#_Toc130148041)

[Supplemental Table 2: Bias assessment 6](#_Toc130148042)

[Supplemental Table 3: Overview of included studies according to children, adults and mixed/unknown age group 7](#_Toc130148043)

[Supplemental Table 4: Dengue characteristics and clinical complications 25](#_Toc130148044)

[Supplemental Table 5: Dengue virus serotypes and pleural effusion 26](#_Toc130148045)

[References 27](#_Toc130148046)

## Supplemental Figure 1: Classification of disease severity


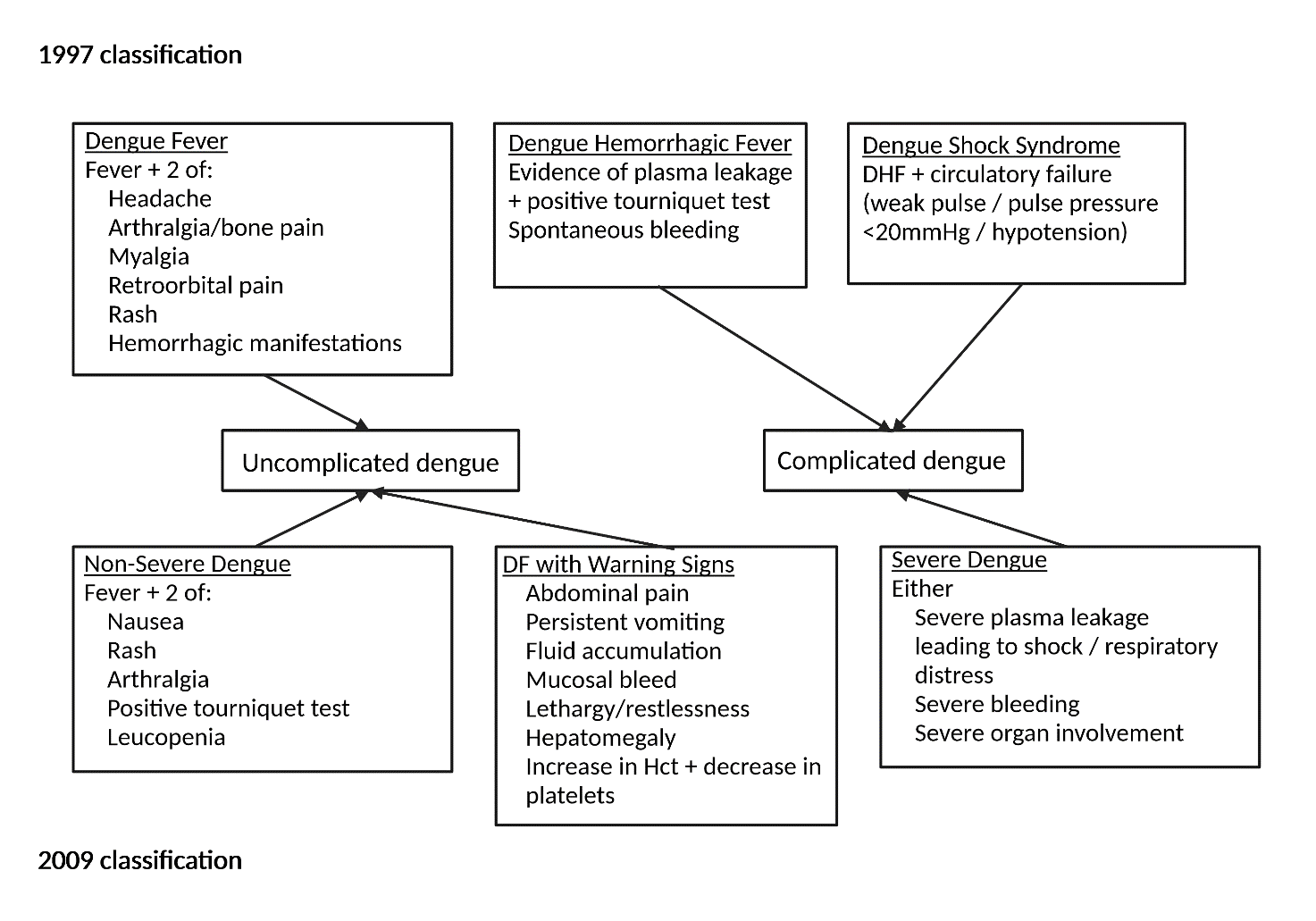
Overview of disease severity classification with reference to the 1997 and 2009 disease classifications by the World Health Organization[1,2]

## Supplemental Figure 2: Geographical distribution of included studies


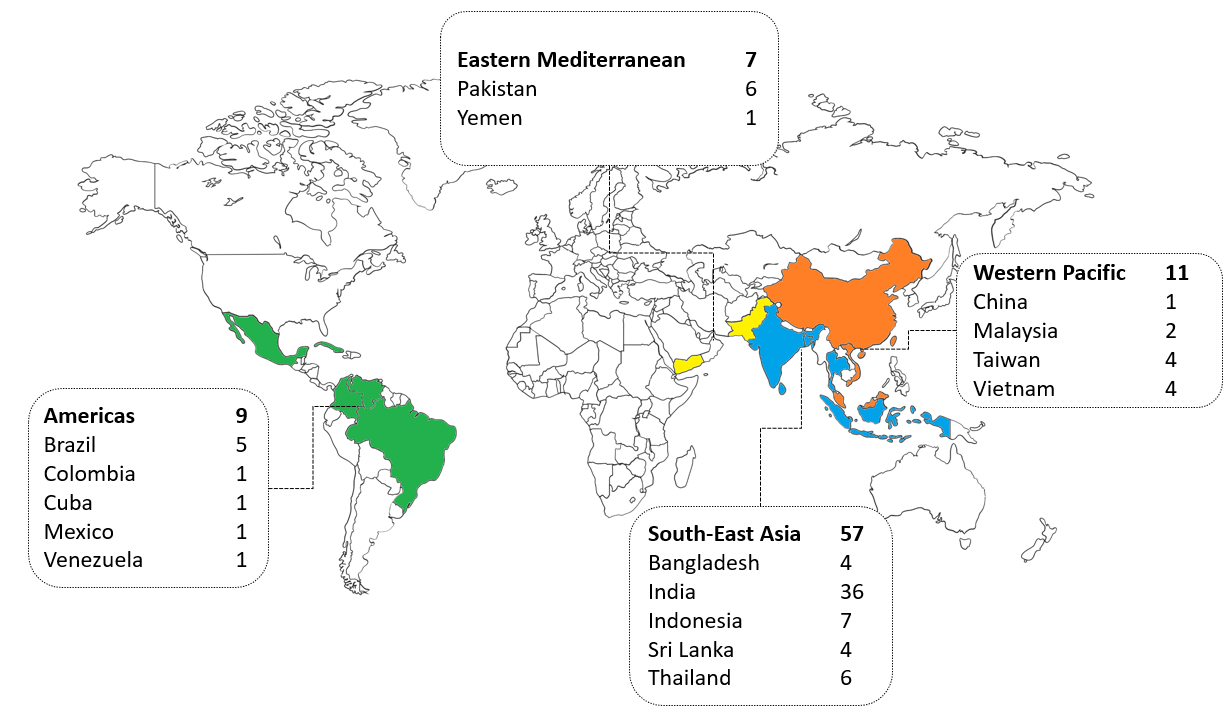


Created with BioRender.com

## Supplemental Figure 3: Frequency of pleural effusion by age and disease severity


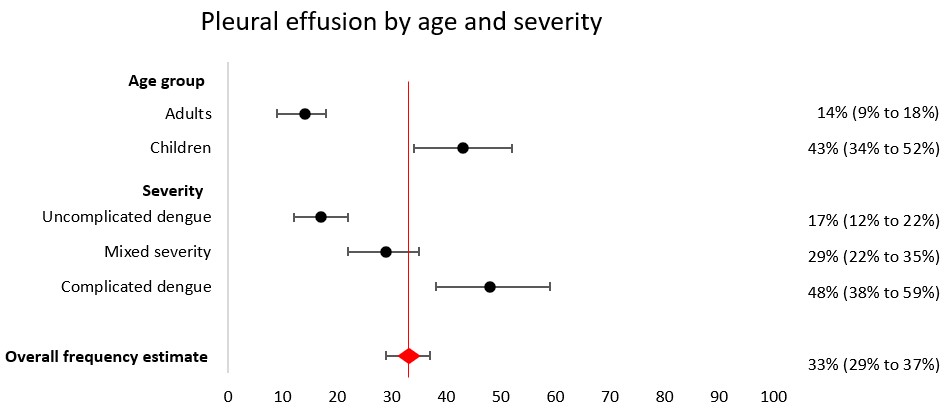


## Supplemental Figure 4: Mechanisms of pleural effusion


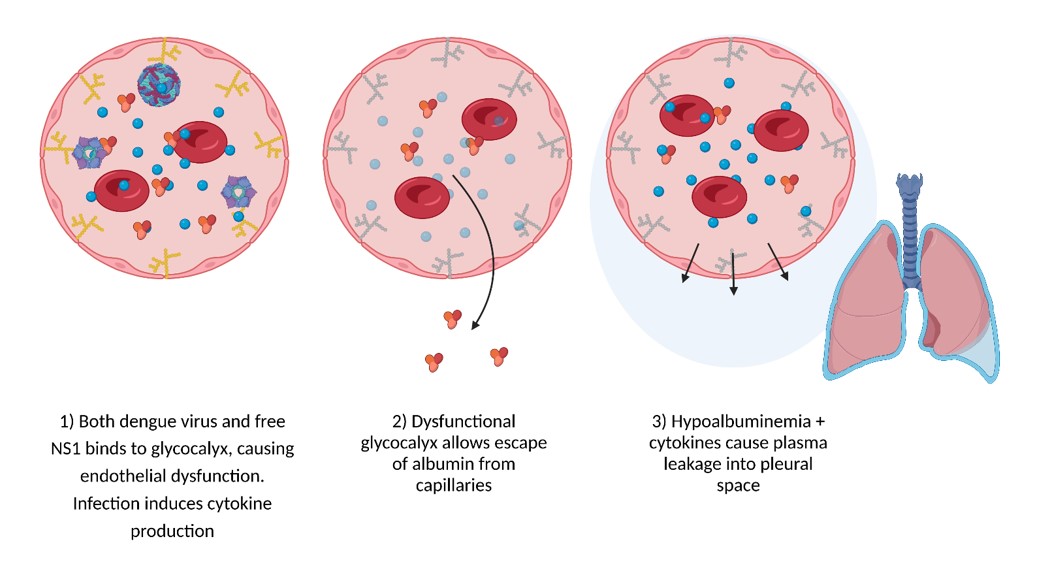


Created with Biorender.com

## Supplemental Table 1: Search strings for databases

| Dengue |  | Symptoms |  | Diagnostics |
| --- | --- | --- | --- | --- |
| **PubMed** |  |  |  |  |
| "Dengue Virus/pathogenicity" [MAJR] OR (((("Severe Dengue/diagnosis" [MeSH]) OR "Severe Dengue/mortality" [MeSH]) OR "Severe Dengue/therapy" [MeSH]) OR "Severe Dengue/virology" [MAJR]) OR "Severe Dengue/epidemiology" [MAJR] OR Dengue infection [Title/Abstract] OR Severe Dengue [Title/Abstract] OR "Severe Dengue/pathology" [MAJR] OR Dengue hemorrhagic [Title/Abstract] OR Dengue shock syndrome [Title/Abstract] OR "Severe Dengue/pathology" [MeSH] | AND | (((((("Hemoptysis/diagnosis" [MeSH]) OR "Hemoptysis/virology" [MAJR]) OR "Lung/virology" [MAJR]) OR Pleural effusion [Title/Abstract] OR "Pulmonary Edema/pathology" [MAJR] OR ARDS [Title/Abstract] OR Acute respiratory distress syndrome [Title/Abstract] OR Diffuse alveolar hemorrhage [Title/Abstract] OR Pulmonary embolism [Title/Abstract] OR pneumonitis [Title/Abstract] OR Lung hemorrhage [Title/Abstract] OR "Hemoptysis/mortality" [MeSH] | AND | "Lung/diagnostic imaging" [MeSH]) OR "Tomography, X-Ray Computed" [MeSH] OR "Predictive Value of Tests" [MeSH]) OR "Prognosis" [MeSH]) OR "Ultrasound" OR "Chest X-ray" OR "Radiography" OR "Pulmonary Edema /diagnostic imaging" [MAJR] |
| **Embase, Web of Science** |  |  |  |  |
| "Dengue" OR "Dengue fever" OR "DENV" OR "Dengue virus" OR "Dengue hemorrhagic fever" OR "Dengue shock" OR "Severe dengue" |  | "Pleural effusion" OR "Pulmonary edema" OR "ARDS" OR "Acute respiratory distress syndrome" OR "Diffuse alveolar hemorrhage" OR "Pulmonary embolism" OR "pneumonitis" OR "Lung hemorrhage" OR "Hemoptysis" OR "Dyspnea" OR "Respiratory distress" OR "Cyanosis" OR "Shortness of breath" |  | "Diagnostic imaging" OR "Imaging" OR "Tomography, X-Ray Computed" OR "CT” "Ultrasound" OR "Chest X-ray" OR "Radiography" OR "Ultrasonography" OR "Magnetic resonance imaging” |
| **Lilacs** |  |  |  |  |
| Dengue OR dengue grave OR virus da dengue OR dengue hemmorrágica OR syndrome do choque da dengue OR dengue grave patologia |  | Hemoptise OR pulmão OR derrame pleural OR edema pulmonar OR ARDS or syndrome de dificuldade respiratoria aguda OR hemmoragia alveolar difusa OR embolia pulmonar OR hemorragia pulmonar |  | Imagem diagnóstica pulmão OR tomografia computadorizada por raios-X OR CT OR ultrasound |

## Supplemental Table 2: Bias assessment

| **Study, year / Raters** | **MDK** | **PB** |
| --- | --- | --- |
| Acharyya et al, 2016[3] | Good | Good |
| Agarwal et al, 2018[4] | Fair | Fair |
| Agarwal & Jain, 2016[5] | Good | Fair |
| Ahlawat et al, 2017[6] | Good | Good |
| Ahmed et al, 2001[7] | Fair | Fair |
| Asghar et al, 2011[8] | Fair | Fair |
| Aziz et al, 2002[9] | Fair | Fair |
| Bajaj et al, 2016[10] | Good | Fair |
| Balasubramanian et al, 2006[11] | Fair | Fair |
| Bandyopadhyay et al, 2016[12] | Good | Fair |
| Bhoi et al, 2014[13] | Poor | Poor |
| Cam et al, 2002[14] | Good | Good |
| Chacko et al, 2008[15] | Poor | Fair |
| Chai et al, 2020[16] | Good | Fair |
| Chandak et al, 2016[17] | Fair | Fair |
| Da Costa et al, 2017[18] | Good | Good |
| Dayananda et al, 2018[19] | Fair | Fair |
| De Kruif et al, 2008[20] | Good | Good |
| Deshwal et al, 2015[21] | Poor | Poor |
| Dhanoa et al, 2016[22] | Good | Good |
| Dhooria et al, 2008[23] | Poor | Poor |
| Djamiatun et al, 2012[24] | Good | Good |
| Ejaz et al, 2011[25] | Fair | Fair |
| Fried et al, 2010[26] | Good | Good |
| Giraldo et al, 2010[27] | Good | Good |
| Godbole et al, 2014[28] | Fair | Fair |
| Gonzalez et al, 2005[29] | Good | Good |
| Gonzalez et al, 2008[30] | Fair | Fair |
| Goyal et al, 2013[31] | Fair | Fair |
| Gupta et al, 2016[32] | Good | Fair |
| Gupta et al, 2020[33] | Good | Fair |
| Hayat et al, 2020[34] | Fair | Fair |
| Hu et al, 2015[35] | Good | Fair |
| Huang et al, 2018[36] | Good | Good |
| Islam et al, 2012[37] | Fair | Fair |
| Jain et al, 2017[38] | Good | Fair |
| Jain et al, 2017[39] | Good | Fair |
| Jain et al, 2020[40] | Good | Fair |
| Joshi et al, 2011[41] | Good | Good |
| Kabra et al, 1992[42] | Poor | Poor |
| Kalayanarooj et al, 1997[43] | Good | Good |
| Kasim et al, 1991[44] | Poor | Poor |
| Khurram et al, 2016[45] | Good | Fair |
| Kuo et al, 2018[46] | Fair | Good |
| Lee et al, 2016[47] | Fair | Good |
| Malavige et al, 2018[48] | Good | Good |
| Manam et al, 2018[49] | Good | Fair |
| Michels et al, 2013[50] | Good | Good |
| Mishra et al, 2016[51] | Good | Fair |
| Mohamed et al, 2013[52] | Fair | Fair |
| Motla et al, 2011[53] | Good | Good |
| Nainggolan et al, 2018[54] | Good | Fair |
| Oliveira et al, 2010[55] | Good | Good |
| Parmar et al, 2017[56] | Good | Fair |
| Parmar et al, 2019[57] | Good | Good |
| Pereira et al, 2018[58] | Good | Good |
| Phung et al, 2019[59] | Fair | Fair |
| Pone et al, 2016[60] | Fair | Fair |
| Pothapregada et al, 2016[61] | Good | Fair |
| Pramuljo et al, 1991[62] | Poor | Poor |
| Prasad et al, 2013[63] | Good | Good |
| Premaratna et al, 2012[64] | Fair | Fair |
| Quiroz-Moreno et al, 2006[65] | Good | Fair |
| Rathi et al, 2015[66] | Good | Fair |
| Rathore et al, 2020[67] | Good | Good |
| Rodrigues et al, 2014[68] | Fair | Fair |
| Sahana et al, 2015[69] | Good | Fair |
| Santhosh et al, 2014[70] | Poor | Poor |
| Schmitz et al, 2011[71] | Good | Good |
| Setiawan et al, 1998[72] | Good | Good |
| Shabbir et al, 2018[73] | Good | Fair |
| Shah et al, 2004[74] | Good | Good |
| Soller et al, 2014[75] | Fair | Fair |
| Srikiatkhachorn et al, 2007[76] | Good | Good |
| Srinivasa et al, 2014[77] | Good | Fair |
| Sudarsi et al, 2016[78] | Fair | Fair |
| Tassniyom et al, 1997[79] | Good | Fair |
| Torres et al, 2004[80] | Good | Good |
| Venkata et al, 2005[81] | Fair | Fair |
| Voraphani et al, 2010[82] | Fair | Fair |
| Weerakoon et al, 2011[83] | Good | Good |
| Wu et al, 2004[84] | Good | Fair |
| Yacoub et al, 2012[85] | Good | Good |
| Yacoub et al, 2017[86] | Good | Good |
| Yousaf et al, 2011[87] | Fair | Fair |
| MDK: Molly Dam Kaagaard; PB: Philip Brainin | | |

## Supplemental Table 3: Overview of included studies according to children, adults and mixed/unknown age group

| **Children** | | | | | | | |
| --- | --- | --- | --- | --- | --- | --- | --- |
| **Author, country, year, design** | **Population** | **Dengue test and severity** | **Symptoms (respiratory)** | **USG** | **CXR** | **Other imaging** | **Outcome** |
| Rathore et al, 2020, Sri Lanka[67]  Prospective | N=84, hospitalized  Children < 12  Mean age 7.9±2.5  M: 61% | NS1 antigen test, RT-PCR  *1997:*  DF: 45 (54%) DHF: 39 (46%)  *2009*:  SD: 16%  Secondary: 62% |  | Pleural effusion: 41 (49%)  - DF: 2 (4%)  - DHF 39 (100%)  Ascites: 30 (36%) |  |  |  |
| Phung et al, 2019, Vietnam[59]  Cross-sectional | N=800, hospitalized (CXR in 135)  Children < 16 yrs  Mean age: 9±3  M: 384 (48%) | NS1 rapid test + MAC ELISA  Only DSS | Dyspnea: 135 (17%)  Respiratory distress: 137 (17%) |  | Pleural effusion:  - PE: 42 (31%)  - PEI <15%: 21/135 (16%)  - PEI 15-30%: 13/135 (10%)  - PEI >30%: 8 (6%) |  | Mortality: 2 (0.3%)  Mechanical ventilation: 8 (1%) |
| Agarwal et al, 2018, India[4]  Retrospective | N=188, hospitalized  Children <12  Mean age: 8 yrs  M: 61% | NS1 ELISA / IgM capture ELISA  DFWS: 131 (70%)  SD: 52 (28%) | ARDS: 3% | Ascites: 10% | Pleural effusion: 10% |  | Mortality: 2 (1%) |
| Pone et al, 2016, Brazil[60]  Retrospective | N=145, hospitalized  Children 2 mths - 18 yrs  M: 68 (47%) | PCR, IgM, sero-conversion, clincal-epidemiological diagnosis (n=48)  NSD: 122 (84%)  SD: 23 (16%)  Secondary: 3 (2%) | Dyspnea: 18 (12%)  Abnormal breath sounds: 60 (41%) |  | Pleural effusion (CXR/USG): 59 (41%)  - NSD: 40 (33%)  - SD: 19 (83%)  P<0.001 |  |  |
| Pothapregada et al, 2016, India[61]  Retrospective | N=261, hospitalized  Children < 13  Mean age: 7±3 yrs  M: 142 (54%) | NS1 / IgM + IgG ELISA  NSD: 159 (61%)  SD: 102 (39%)  Secondary: 17 (7%) |  | Pleural effusion: 13 (5%)  Ascites: 18 (7%) |  |  | Mortality: 6 (2%) |
| Rathi et al, 2015, India[66]  Prospective | N=100, hospitalized  Children 9 mo- 18 yrs  Mean age: 12 yrs  M: 72 (72%) | NS1, IgM and/or IgG (rapid diagnostic kit)  NSD: 52 (52%)  DFWS: 40 (40%)  SD: 8 (8%)  Secondary: 26 (26%) | Respiratory distress: 5 (5%) | PE: 9 (9%)  Ascites: 16 (16%) | PE: 20 (20%)  - Bilateral: 4 (4%) |  | Mortality 3 (3%) - 2 from ARDS |
| Sahana & Sujatha, 2015, India[69]  Prospective | N=81  Children  M: 55 (68%)  Mean age: 8 yrs | NS1 immuno-  chromatography test / IgM + IgG immunochroma-  tography  NSD: 39 (48%)  DFWS: 22 (27%)  SD: 20 (25%)  Secondary: 15 (19%) |  | Ascites: 39 (48%) | PE: 32 (40%)  - NSD: 1 (3%)  - DFWS: 16 (73%)  - SD: 18 (90%) |  | Mortality: 2 (3%) |
| Soller et al, 2014, Thailand[75]  Prospective | N=19, hospitalized  Children 0.5-15 yrs  Mean age: 10±3 yrs  M: 13 (68%) | RT-PCR / ELISA  DF: 8 (42%)  DHF: 8 (42%)  DSS: 3 (16%) |  | PE: 9 (47%)  - Admission: 2 (11%)  - Fever day -1: 3/9 (33%)  - Fever day 0: 6/17 (35%)  - Fever day 1: 9/17 (53%)  - Fever day 2: 4/9 (44%) | Mean PEI (CXR) fever day 1 = 21 |  |  |
| Srinivasa et al, 2014, Bangladesh[77]  Cross-sectional | N=200, hospitalized  Children  M: 106 (53%) | BioLine NS1 / IgM + IgG  DSS: 47 (24%) |  | Pleural effusion: 93 (47%)  - DSS: 47 (100%)  Ascites: 74 (37%) |  |  | Mortality: 4 (2%)  (shock + ARDS) |
| Prasad et al, 2013, India[63]  Prospective | N=56, hospitalized (n=51) + outpatient (n=5)  Children 2-12 yrs  Mean age: 6±3 yrs  M: 41 (73%) | ELISA IgM / RT-PCR / NS1  DHF/DSS: 13 (23%) / SD: 33 (59%) | Respiratory distress: 13 (23%) | Ascites: 4 (7%) | PE: 8 (14%)  - DF: 6 (11%)  - DHF/DSS: 2 (15%)  - SD: 8 (24%) |  | Mortality: 10 (20%) |
| Djamiatun et al, 2012, Indonesia[24]  Prospective | N=73, hospitalized for DHF + 17 healthy controls  Children 3-14  Mean age 8 (6-9) for DHF, 7 (6-9) for DSS  M: 25 (34%) | IgM + IgG capture and indirect ELISA  DHF: 43 (58%)  DSS: 30 (42%) |  |  | PE:  - Day 0: 48/59 (81%)  - Day 2: 63/63 (100%)  PEI:  - DHF, day 0: 10 (2-20)  - DSS, day, 0: 19 (13-29)  - DHF, day 2: 25 (15-33)  DSS, day 2: 31 (23-43) |  | Mortality: 6 (8%) |
| Joshi & Baid, 2011, India[41]  Retrospective | N=57, hospitalized, children - 3 mths - 15 yrs  M: 34 (60%)  Mean age: 5±4 yrs | IgM ELISA  DF: 28 (49%)  DHF: 11 (19%)  DSS: 18 (32%) | Pulmonary hemorrhage: 3 (14%) | Ascites: 9 (16%) | Pleural effusion: 18 (32%) |  | Mortality: 2 (4%)  Mechanical ventilation: 5 (9%) |
| Fried et al, 2010, Thailand[26]  Prospective | N=457, outpatient + hospitalized  Children 18 mths - 15 yrs  Mean age: 9±3 yrs  M: 254 (56%) | IgG/IgM HAI / RT-PCR / virus isolation by mosquito inoculation  DF: 289 (63%) DHF: 169 (37%)  Secondary: 357 (78%) |  |  | PE: 167 (37%) - Primary: 15 (9%) - Secondary: 152 (91%)  Mean PEI: 5.7  Ascites: 63 (14%) |  |  |
| Giraldo et al, 2010, Brazil[27] | N=181, hospitalized  Children <15  Mean age: 9±4 yrs  M: 88 (49%) | Serology - unspecified  DF: 94 (52%)  DHF: 87 (48%) | - Respiratory distress: 16 (9%)  - Diminished breath sounds: 19 (10%) |  | PE (CXR/USG): 37/181 (20%)  - DF: 7/94 (7%)  - DHF: 30 /87 (34%)  P=0.015  Pleural opacities: 24 /181 (14%)  Atelectasis: 4/181 (2%)  Pulmonary edema: 2/181 (1%)  Ascites: 13 (7%) |  | Mortality: 0  Ventilatory support: 7 (4%) - all PM, 6 DHF  Thoracentesis: 1 (0.6%) |
| Oliveira et al, 2010, Brazil[55]  Prospective | N=37  Children  M: 19 (51%)  Mean age: 8 yrs (range 0.1-17) | Serology  Only DHF/DSS |  | PE: 26 (70%) at 1. examination, all had resolved at 2.  Ascites: 29 (78%) |  |  |  |
| Voraphani et al, 2010, Thailand[82]  Prospective | n=27, Hospitalized children | ELISA  DF: 17 DHF: 10 |  | PE: 5 (19%) |  |  |  |
| de Kruif et al, 2008, Indonesia[20]  Prospective | n=56, PICU  Children 3-14 yrs  Median age: 6 yrs (5-9)  M: 22 (39%) | ELISA IgM + IgG / NS1 dot blot immuno-assay / RT-PCR  DF: 7 (13%)  DHF: 29 (51%)  DSS: 20 (36%)  Secondary: 52 (93%) |  | Pulmonary edema: 1 (2%) | PE (CXR/ USG): 44 (79%)  - DF/DHF: 26 (72%)  - DSS: 18 (90%)  Ascites: 19 (34%) |  | Mortality: 4 (7%) |
| Chacko et al, 2008, India[15]  Prospective | N = 73  Children - mean age 7.9  M: 44  Hospitalized | ELISA  *2009* NSD: 24 (33%) SD: 49 (67%) *1997* DSS: 34 (47%) |  | PE: 22/44 (50%)  - DSS: 17 | PE: 21/59 (36%)  - DSS: 15 |  | Mortality: 2 (3%) |
| Dhooria et al, 2008, India[23]  Retrospective | N=81, hospitalized for DHF  Children, 91% <6 yrs  M: 53 (65%) | IgM  DHF: 75 (93%)  DSS: 6 (7%) | Respiratory distress: 6 (7%)  Respiratory failure: 3 (4%)  ARDS: 2 (2%)  (All undefined) | Ascites: 0 | Pleural effusion: 9 (11%) - 2 with ARDS |  | Mortality: 3 (4%)  2 ARDS  Mechanical ventilation: 3 (4%) |
| Srikiatkhachorn et al, 2007, Thailand[76]  Prospective | N=82, hospitalized + 76 OFI-controls  Children <15 yrs  Mean age: 10±0.5  M: 38 (46%) | RT-PCR, ELISA, HAI  DF: 61 (74%)  DHF: 16 (20%)  DSS: 5 (6%)  Secondary: 71 (87%) |  | PE:  *Admission*  - OFI: 0  - DHF: 2 (10%)  *Fever day -1*  - OFI: 0  - DF: 1 (2%)  - DHF/DSS: 3 (16%)  *Fever day 0*  - DHF/DSS: 7 (33%)  *Fever day 1*  - DF: 1 (2%)  DHF: 8 (50%)  - DSS: 5 (100%) -  - OFI: 0% | PE:  *Fever day 1*  - DF: 5 (9%)  - DHF/DSS: 21 (100%)  - OFI: 8 (19%)  PEI - fever day 1:  - DF: 0.2±0.1%  - DHF/DSS: 16±3%  - OFI: 0.4±0.1% |  |  |
| Balasubramanian et al, 2006, India[11]  Prospective | N = 65 Children (>1month-18 yrs)  Hospitalized | ELISA IgM + IgG  DF: 30  DHF: 35 (including 6 DSS) |  | PE: 44 (68%)  - DF: 13 (68%)  - DHF: 31 (89%)  Ascites: 46 (71%) | PE: 29  - DF: 6 (20%)  - DHF: 23 (66%) |  |  |
| Venkata et al, 2005, India[81]  Retrospective | N=88 + 40 controls  M: ?  Mean age: 5 yrs (range 2-9) | Serologically – unspecified  DF: 65  DHF: 19  DSS: 4 |  | Pleural effusion (included on day 2-3 of fever (n=32):  - Day 2-3: 2/32 (6%)  - Day 5-7: 30/32 (94%)  Included day 5 (n=56)  - Day 5-7: 55 (96%)  Ascites: 21 (24%) |  |  |  |
| Shah et al, 2004, India[74]  Prospective | N = 39, hospitalized  Children  Mean age: 4.9 yrs, range 0.3-12 | IgM ELISA  DF: 1  DHF: 20  DSS: 18 | Cough: 15 (39%) | Ascites: 14 (36%) | PE: 21 (31%) - all with ascites |  | Mortality: 3 (8%) |
| Cam et al, 2002, Vietnam[14]  RCT | N = 37  Hospitalized – ICU All DSS  Children <15 yrs, mean (range) 6 (1-11)  M: 12 (32%) | IgM+IgG ELISA, HAI | PaO_2_: 91 mmHg (O_2_ mask), 64.5 mmHg (NCPAP) |  | PE: 34 (92%) |  | Mortality: 4  Pleural puncture needed: 2 |
| Ahmed et al, 2001, Bangladesh[7]  Prospective | N=72, hospitalized  Children<12  Mean age: 8.4±3 yrs  M: 42 (58%) | ELISA DOT IgM / IgG  DF: 26 (36%)  DHF: 36 (50%)  DSS: 10 (14%) | Cough: 3 (4%)  Respiratory difficulty: 4 (6%) - all DSS | Ascites: 1 (2%) - DHF | PE: 2 (3%) - 1 DSS, 1 DHF |  | Mortality: 5 (7%) |
| Setiawan et al, 1998, Indonesia[72]  Prospective | N=148  M: 75  5 mo. - 14 yrs | HAI or ELISA for IgM + IgG / virus  isolation in a tube culture of C6/36 cells  DHF: 73  DSS: 75 |  | PE: 93 (63%)  - DHF: 22 (30%)  - DSS: 71 (95%)  Bilateral: 63 |  |  |  |
| Kalayanarooj et al, 1997, Thailand[43]  Prospective | N = 60 dengue  Hospitalized (for observation for the study)  32 M, mean age 8.1 yrs ± 3 | ELISA / HAI for IgM + IgG  93% 2°  DF: 32  DHF: 28 |  |  | PE:24/56 (43%) - DF: 2/30 (7%) - DHF: 22/26 (84%) |  |  |
| Tassniyom et al, 1997, Thailand[79]  RCT | N=95 (50 placebo), hospitalized  Children  M: 46 (48%)  Mean age: 7.3±3 (intervention), 8.3±3 (placebo) | HAI IgM  DSS: 7 (7%)  Secondary: 77 (81%) | Cough: 20 (21%) - 10 in each |  | PE: 30 (32%) - 15 in each group |  |  |
| Kabra et al, 1992, India[42]  Prospective | N=15, children, severe  6-12 yrs | IgM HAI / ELISA  DHF: 1  DSS: 14 |  | Ascites: 15 (100%) | PE: 13 (87%) |  | Mortality: 2 (13%) |
| Kasim et al, 1991, Indonesia[44]  RCT | N=85, hospitalized  Children  M: 34 (40%) | HAI - positive in 64 (76%)  DHF: 45 (53%)  DSS: 40 (47%) | Hyper-ventilation: 74 (87%)  Hypoxemia:  31 (36%) |  | PE: 42 (49%)  Pulmonary vascular engorgement: 28 (33%) |  |  |
| Pramuljo et al, 1991, Indonesia[62]  Prospective | N=25, children, DHF  2-14 yrs | IgM/IgG HAI  DSS: 25 |  | Ascites: 18 (72%)  PE: 25 (100%) |  |  | Mortality: 0 |
| **Adults** | | | | | | | |
| **Author, country, year** | **Population** | **Dengue test and severity** | **Symptoms (respiratory)** | **USG** | **CXR** | **Other imaging** | **Outcome** |
| Chai et al, 2020, Malaysia[16]  Prospective | N=83, outpatient, hospital (N=65)  Adults > 18  Mean age: 35±14 yrs  M: 47 (57%) | NS1 / IgM + IgG - unknown method  DFWS in 60 (72%)  SD in 5 (6%)  Secondary infection: 3 (4%) |  | *At presentation:*  PE: 3 (4%)  *Day 2:*  PE: 4 (5%)  *Day 3*:  PE: 1 (2%)  *Day 4:*  PE: 0  *Last follow up:*  PE: 1/18 (5%) |  |  | Mortality: 0 |
| Jain et al, 2020, India[40]  Cross-sectional | N=100 dengue + 100 malaria (vivax), hospitalized, adults  M: 61 (61%)  Mean age: 26 yrs | IgM (ELISA) | Dyspnea:  - DF: 20 (20%)  - Malaria: 4 (4%)  - P<0.05 | Ascites:  - DF: 49 (49%)  - Malaria: 2 (2%)  - P<0.05 | PE:  - DF: 46 (46%)  - Malaria: 2 (2%)  - P<0.05 |  |  |
| Kuo et al, 2018, Taiwan[46]  Retrospective | N=669  Adults >18 yrs  Mean age: 50±17 yrs  M: 341 (51%) | RT-PCR, NS1, IgG sero-conversion  NSD: 320 (48%)  DFWS: 322 (48%)  SD: 27 (4%) | Hemoptysis, febrile phase: 3/338 (1%) |  | PE, febrile phase (CXR/USG): 35/259 (10%)  PE, Critical phase (CXR/USG): 21/186 (11%)  PE, recovery phase (CXR/USG): 7/57 (12%)  Ascites, febrile phase: 4/65 (6%) |  | Mortality: 2 (0.3%) |
| Huang et al, 2018, Taiwan[36]  Retrospective | N = 1300  Adults  Mean age: 50.1±15.5 yrs  M: 606 (47%) | PCR, 4x rise in IgG, NS1  DHF: 354 (27%)  DSS: 30 (2%) |  | Ascites:  124 (10%) | PE (CXR/USG):  181 (14%) |  | In-hospital mortality: 12 (1%) |
| Malavige et al, 2018[48]  RCT | N = 133, hospitalized  18-60 yrs  Age: 34 ± 11  M: 104 (78%) | NS1 test - unspecified  Primary: 32 (24%)  Secondary: 89 (67%) |  | PE: 12 (9%)  Ascites: 33 - Mild: 13 - Moderate: 12 |  |  |  |
| Nainggolan et al, 2018, Indonesia[54]  Prospective | N=69, hospitalized  M: 36 (52%)  Mean age: 24±10 yrs | RT-PCR, NS1  Secondary: 46 (67%) |  | PE:  - 3rd day of fever: 2 (3%)  - At some point: 10 (15%)  Ascites: 19 (28%) |  |  |  |
| Pereira et al, 2018, India[58]  Retrospective | N=550  M: 405 (74%)  Mean age: 32±12 | NS1 ag-test / IgM ELISA  NSD/ DFWS: 449 (82%) SD: 101 (18%) | Dyspnea: 13 (2%) | PE: 13 (2%)  - NSD: 8 (2%)  - SD: 5 (5%)  - P=0.038  Ascites: 44 (8%)  - NSD: 27 (6%)  - SD: 17 (17%) |  |  | Mortality: 7 (1%)  ICU: 54 (10%) |
| Gupta et al, 2016, India[32]  Cross-sectional | N=45, hospitalized + outpatient  Adults > 18  M: 28 (62%)  COPD/astma: 3 (7%) | IgM MAC-ELISA | - Cough: 8 (18%)  - Dyspnea: 5 (11%)  - Hemoptysis: 0  - Pneumonia: 5 (11%)  - Respiratory failure: 7 (16%) | Ascites: 2 (4%) | Pleural effusion: 3 (7%) |  | ICU: 6 (13%)  Mortality: 2 (4%) |
| Lee et al, 2016, Taiwan[47]  Retrospective | N = 1253, hospitalized (646 evaluated for PE)  Mean age 51 (18-93)  M: 47% of total | RT-PCR / NS1 / seroconversion  *2009* SD: 69 (6%)  *1997*  DHF: 146 (12%)  DSS: 26 (2%) | - Cough: 268  - Pneumonia: 4 of 69 SD (6%) | Ascites: 39 (3%) | Pleural effusion (CXR/USG): 79 (12%) - NSD 45/596 (8%) - SD: 16/50 (32%) - DSS: 12 (46%) |  | Mortality: 15 (1%) - all SD  Mechanical ventilation: 22 (32%) of SD |
| Deshwal et al, 2015, India[21]  Prospective | N=515  >12, hospitalized/ outpatient  M: 375 (73%)  21-40 yrs: 324 (63%) |  | Dyspnea: 27 (5%) | Ascites: 84 (16%) | PE: 103 (20%) |  | Mortality: 4 (0.8%) - all secondary DF,  2 DSS, 1 fluid overload |
| Hu et al, 2015, China[88]  Retrospective | N=38, hospitalized | RT-PCR / unspecified IgM test  Only SD |  |  | Pleural effusion: 12/76 (62%)  Patchy exudation: 14/76 (18%)  Atelectasis: 7/76 (9%) | CT / HRCT:  - Pleural effusion: 21/34 (62%)  - Patchy exudation: 20/34 (59%)  - Atelectasis: 18/34 (53%)  Ascites: 4/21 (19%) |  |
| Godbole et al, 2014, India[28]  Retrospective | N=63  M: 53 | NS1 / IgM /IgG sero-conversion ELISA  DF: 60  DHF: 3  DSS: 0 |  | Ascites: 2 (3%) | Pleural effusion: 2 (3%) |  |  |
| Gonzales et al, 2005, Cuba[29]  Retrospective | N=76, hospitalized  Adults 16-64  M: 51 (67%) | IgM-capture ELISA  DHF: 58 (76%)  DSS: 18 (24%) | - Cough: 9 (12%)  - Dyspnea: 7 (9%)  - Chest pain: 8 (11%)  - Pleural effusion (clinical): 6 (8%) | Pleural effusion: 11/54 (20%)  Ascites: 1/54 (2%) |  |  | Mortality: 2 (3%) - DSS |
| Wu et al, 2004, Taiwan[84]  Prospective | N=65, emergency dep  M: 29 (45%)  Mean age: 49±12 yrs - range 18-76 | ELISA IgM / IgG sero-conversion |  | Ascites: 24 (37%)  PE: 21 (32%)  - Bilateral: 11 |  |  | Mortality: 0 |
| **Mixed/unknown age** | | | | | | | |
| **Author, country, year** | **Population** | **Dengue test and severity** | **Symptoms (respiratory)** | **USG** | **CXR** | **Other imaging** | **Outcome** |
| Gupta et al, 2020, India[33]  Prospective | N=50, ICU  M: 35 | MAC ELISA / NS1  DF: 20  DHF: 22  DSS: 8 | - ARDS: 11  - Hemoptysis: 15  - Dyspnea: 28 | PE: 7 (14%)  - DF: 2 (10%)  - DHF: 4 18%)  - DSS: 1 (13%) |  |  | Mortality: 27 |
| Hayat et al, 2020, Pakistan[34]  Retrospective | N=171 (only those with imaging included - 44% of total)  M: 117 (68%)  Mean age: 36 yrs (range 0.4-86) | Serology |  | Ascites: 73 (43%)  PE: 90 (53%)  - Mild: 85 (94%)  - Moderate, right: 5 (6%)  - Severe: 1 (1%) |  |  |  |
| Parmar et al, 2019, India[57]  Prospective | N=84, severe dengue  Children + adults (1-81 yrs) | NS1 / IgM + IgG |  | PE: 53 (63%)  Ascites: 67 (80%) |  |  | Mortality: 0 |
| Dayananda og Halawar, 2018, India[19]  Prospective | N=110 suspected dengue + referred to USG - DF: 67 (61%), non-DF: 43 (39%)  M: 76 (69%)  Mean age: 41 yrs (range 2-80)  <18 yrs, DF: 32/67 (48%) | Rapid solid phase  Immuno-chromato-graphic test NS1 / differential detection of IgM + IgG |  | PE:  DF, total: 39 (58%)  - DF, <18: 21 (67%)  - DF, >18: 18 (51%)  - non-DF: 31 (72%) |  |  |  |
| Manam et al, 2018, India[49]  Prospective | N=378, hospitalized  M: 224 (59%)  Mean age: 34±3, range 6-64 | Dengue card test for IgM + IgG / NS1  DHF: 15 (12%) |  | PE: 186 (49%)  - Bilateral: 102 (27%)  - Right: 68 (18%)  - Left: 16 (4%)  - 0-9 yrs: 23/68 (34%)  - 10-29 yrs: 65/124 (52%)  - 30-49 yrs: 43/108 (40%)  - >49 yrs: 55/78 (71%) |  |  |  |
| Mishra et al, 2018, India[51]  Prospective | N=96  Range: 14-75 yrs  Age group 31-60: 59 (64%)  M: 61 (64%) | NS1, IgM  DFWS: 14 (15%)  SD: 3 (3%) | - Cough: 8 (8%)  - Shortness of breath: 3 (3%)  - ARDS: 0 | Ascites: 8 (8%) | PE: 8 (8%) |  | Mortality: 0 |
| Shabbir et al, 2018, Pakistan[73]  Cross-sectional | N=79  Mean age: 37±12  M: ? | NS1 ELISA / PCR  Only DF+DHF |  | Pleural effusion: 10  - Right: 4  - Left: 5  - Bilateral: 1 |  |  | Mortality: 0 |
| Ahlawat & Kalra, 2017, India[6]  Prospective | N=61, hospitalized  M: 40 (66%)  Mean age: 26.5±12  <15 yrs: 9 (15%) | ELISA IgM / PCR | ARDS: 1 (1.6%) | PE: 10 (16%)  Ascites: 13 (21%) |  |  | Mortality: 0 |
| da Costa Faria et al, 2017, Brazil[18]  Prospective | N=94, hospitalized (27) + outpatient  Mean age: 34 | MAC-ELISA / NS1-ELISA / PCR  DFWS: 29 (31%)  SD: 4 (4%) |  |  | PE: 4 (4%) |  |  |
| Jain et al, 2017, India[39]  Prospective | N=369, hospitalized, >14 yrs  Mean age: 31±14 yrs  M: 249 (68%) | Dengue diagnostic kit for NS1 / IgM  DF: 198 (54%)  DHF:125 (34%)  DSS: 46 (12%) | - Crepitations on chest auscultation: 21 (6%)  - Dyspnea: 29 (8%)  - Chest pain: 5 (1%)  - ARDS: 14 (4%) - all DSS |  | PE: 180 (49%)  - DF: 91 (46%)  - DHF: 68 (54%)  - DSS: 21 (50%)  - Bilateral: 126 (34%)  - Right: 47 (13%)  - Left: 7 (2%) |  | Mortality: 22 (6%)  Assisted ventilation: 27 (7%) |
| Jain et al, 2017, India[38]  Cross-sectional | N=101, in Emergency department, >14 yrs  M: 64 (63%) | Dengue diagnostic kit for NS1 + IgM  DF: 41 (41%)  DHF: 52 (51%)  DSS: 8 (8%) | - Dyspnea: 6 (6%)  - Hemo-ptysis: 2 (2%)  - ARDS: 3 (3%) | Ascites: 13 (13%) | Pleural effusion (CXR/USG): 10 (10%) |  | Mortality: 3 (3%) |
| Parmar et al, 2017, India[56]  Prospective | N=93 dengue + 67 dengue-like  Age 1-77 yrs | Lateral flow immuno-  chromatography rapid test for NS1 / ELISA for IgM + IgG |  | Ascites: 40 (43%) with dengue + 9 (13%) without  PE: 70  - Bilateral: 18 |  |  |  |
| Yacoub et al, 2017, Vietnam[86]  Prospective | N=102, hospitalized - ICU / severe dengue  Median age: 11 (IQR 8-14)  M: 54 (53%) | Capture ELISA IgM + IgG / RT-PCR  DHF: 22 (22%)  DSS: 80 (78%) | Respiratory distress: 19 (19%)  Day 2: 10 (53%)  Day 3: 8 (42%)  Day 4: 1 (5%) |  | Pleural effusion: 19 (19%)  Pulmonary edema: 0 |  | Mortality: 1 (1%) |
| Acharyya et al, 2016, India[3]  Cross-sectional | N=382, hospitalized  M: 259 (68%)  Mean age: 38±18 yrs, range 1-81 | NS1 ELISA / IgM ELISA (MAC-ELISA) / IgG ELISA / HAI  DSS: 24 (6%) |  | Ascites: 43 (11%)  Pleural effusion: 101 (26%) | Pulmonary edema: 10 (2.6%) |  | Mortality: 7 (2%) |
| Agarwal et Jain, 2016, India[5]  Prospective | N=126  Age >15 | IgM / IgG ELISA |  | PE: 61 (48%)  Ascites: 81 (64%) |  |  |  |
| Bajaj & Chander, 2016, India[10]  Cross-sectional | N=178, hospitalization unknown  Children + adults  M: 117 (66%) | NS-1 Ag test /  dengue IgG / IgM test |  | Ascites: 98 (55%)  Pleural effusion: 32 (18%)  - Unilateral: 12 (7%)  - Bilateral: 20 (11%) |  |  |  |
| Bandyopadhyay et al, 2016, India[12]  Prospective | N=110  M: 62  Age range: 14-62 | NS1 ELISA / IgM MAC ELISA  DF: 59  DHF: 26  DSS: 25 |  | PE: 55 (50%)  - DF: 19 (32%)  - DHF: 19 (73%)  - DSS: 17 (68%)  Ascites: 58 |  |  |  |
| Chandak & Kumar, 2016, India[17]  Prospective | N=107 with radiological findings of 400 confirmed dengue  M: 46  79% 20-50 yrs, range 1-82 | NS1 rapid solid-phase immune-chromatographic test / IgM detection |  | USG (N=85):  PE (total): 53 (62%)  - PE (unilateral): 34 (40%)  - PE (bilateral): 19 (22%)  Ascites: 31 (36%) | CXR (n=21):  PE (bilat): 1 (5%) |  |  |
| Dhanoa et al, 2016, Malaysia[22]  Retrospective | N=262, hospitalized  M: 146 (56%)  Median age: 28 yrs (range 3-75)  Pediatric: 38 (15%) | NS1 rapid dengue diagnostic kit / PCR + IgG Capture ELISA  SD: 17 (6%)  Secondary: 82 (31%) | - Cough: 30 (12%)  - Tachypnea: 32 (12%)  - Respiratory distress: 7 |  | Pleural effusion: 6 (2%) |  | Mortality: 1 (0.4%)  ICU: 4 (2%)  Mechanical ventilation: 4 (2%) |
| Khurram et al, 2016, Pakistan[45]  Prospective | N=240, hospitalized, only DHF  M: 166 (69%)  Mean age: 29±12 yrs | NS1, IgM, sero-conversion  DHF: 229 (95%) DSS: 11 (5%) |  | Pleural effusion: 110/215 (51%)  - Unilateral: 83 (39%)  - Bilateral: 27 (13%)  - Mild: 98 (46%)  - Moderate: 12 (6%)  - DHF: 103/229 (45%)  - DSS: 7/11 (64%) |  |  | Mortality: 8 (3%) |
| Sudarsi et al, 2016, India[78]  Prospective | N=50, hospitalized  >12 yrs  M: 33 (66%) | IgM, unknown method  DF: 28 (56%)  DHF: 18 (36%)  DSS: 4 (8%) | ARDS: 2 (4%) - all DSS |  | PE: 11 (22%)  - DF: 1 (4%)  - DHF: 8 (44%)  DSS: 2 (50%) |  | Mortality: 3 (6%) |
| Bhoi et al, 2014, India[13]  Prospective | N = 21, Hospitalized  M: 16  Mean age: 33±19 (range 5-69) yrs | ELISA NS1 + IgM  DSS: 3 |  |  | Ascites: 4 (19%)  PE: 4 (19%) |  | Mortality: 1 (5%)  Mechanical ventilation: 4 (19%) |
| Rodrigues et al, 2014, Brazil[68]  Retrospective | N = 2020 DF - of these, 29 (1.4%) with CT-scans were included  Hospitalized/ outpatient  >16 yrs  M: 18 (62%)  Median age: 58 yrs (range 16-90) | IgM - unknown method  NSD: 9 (31%)  SD: 20 (69%)  Secondary: 10 | - Dyspnea: 11 (38%)  - Cough: 8 (28%) |  |  | CT:  - Pleural effusion: 16 (55%) - 5 NSD, 11 SD  - Ground glass opacities: 8 | Mortality: 4  ICU: 17 (59%) |
| Santhosh et al, 2014, India[70]  Retrospective | N=96 – all patients referred for USG  M: ?  Age: ? | NS1, IgM - unknown method |  | Pleural effusion: 48 (50%)  - Bilateral: 25 (26%)  Ascites: 62 (65%) |  |  |  |
| Goyal et al, 2013, India[31]  Prospective | N=426, outpatient + emergency department  Range 11-80  M: 236 (55%) | Dengue serology NS1, IgM, IgG |  | PE (USG/CXR): 22 (6%) |  |  | Mortality: 0 |
| Michels et al, 2013, Indonesia[50]  Prospective | n= 66, hospitalized  Mean age 21 years  58% male | RT-PCR, IgM + IgG HAI  SD: 11 (17%) | Respiratory rate: 20-22 | PE: 2 (3%)  - NSD: 2 (4%)  - SD: 0 (0%) |  |  |  |
| Mohamed et al, 2013, Yemen[52]  Prospective | N=100, hospitalized  M: 54 (54%)  <50 yrs: 65% | MAC-ELISA / PCR  DF: 43 (43%)  DHF: 42 (42%)  DSS: 15 (15%) | - Cough: 38  - Dyspnea: 57  - ARDS: 16  - Hemoptysis: 32 |  | Pulmonary hemorrhage (CXR/CT): 10 (10%)  PE (CXR/CT): 3 (3%)  - DF: 0  - DHF: 3 (7%)  - DSS: 0  P=0.12 |  | Mortality: 55 (55%) |
| Islam et al, 2012, Bangladesh[37]  Prospective | N=50  Median age: 60 yrs, range: 16-65 | IgM ELISA  DF: 25 (50%)  DHF: 25 (50%) | Hemoptysis: 1 (2%) – DHF  Respiratory distress: 1 (2%) - DF |  | Ascites: 1 (2%)  PE: 1 (2%) - DHF |  | Mortality: 0 |
| Premaratna et al, 2012, Sri Lanka[64]  Prospective | N = 102, hospitalized  M: 52 (51%)  Children/ adults (>12 yrs)  Mean age: 28.3±12 yrs | DHF: 41 (40%)  DSS: 3 (3%) |  | Pleural effusion: 33 (32%)  Ascites: 21 (21%) |  |  |  |
| Yacoub et al, Vietnam, 2012[89]  Prospective | n=79, hospitalized  Median age 20 years (range 8-46 yrs)  M: 50 (63%) | IgM+IgG ELISA, NS1 assays  *2009* DF: 22  DFWS: 42  SD: 15  *1997* DF: 1  DHF: 65 DSS: 13 | RR 20-22 | PE: 25 (32%)  NSD: 1 (5%)  DFWS: 12 (30%)  SD: 12 (92%) |  |  |  |
| Asghar & Farooq, 2011, Pakistan[8]  Retrospective | N=76, with imaging results, hospitalized (of 500)  M: 43 (57%)  Mean age: 39 yrs, range 14-80 | HAI / ELISA / Virus isolation in culture of C6/36 cells  All DHF |  |  | USG/CXR:  Ascites: 39 (51%)  Pleural effusion: 24 (32%) |  |  |
| Ejaz et al, 2011, Pakistan[25]  Cross-sectional | N=663, hospitalized (CXR in 299)  Mean age of those with PE: 32±15  M: 31 (62%) | IgM or unknown  DF: 43/50 (96%)  DHF: 1/50 (2%)  DSS: 1/50 (2%) | No respiratory complaints: 354 (53%)  Dyspnea: 2/50 (4%) |  | Pleural effusion: 50/299 (17%):  - Right: 23/50 (46%)  - Hereof 1 DHF  - Left: 9/50 (18%)  - Bilat: 18/50 (36%)  - Hereof 1 DSS |  |  |
| Motla et al, 2011, India[53]  Retrospective | N=73 dengue + 96 febrile without dengue, all hospitalized  Children + adults 3-75 yrs  Mean age all: 28±13  M: 56 (77%) | IgM + IgG - unknown method  Secondary: 34 (47%) |  | Ascites:  - DF: 65/73 (89%)  - OFI: 61/96 (64%)  PE:  - DEN: 40 (54%)  - OFI: 27 (28%) |  |  |  |
| Schmitz et al, 2011, India[71]  Retrospective | N = 43 DHF/DSS  ICU-patients  Adults (>15 yrs)  Median age 38 yrs  M: 66% | Duo IgM and IgG rapid strip test  DHF/DSS: 43  Secondary: 36 (84%) | Respiratory failure: 13 (30%)  - Survivors: 4 (12%)  - Non-survivors: 7 (88%)  P<0.001 | Ascites:  35/42 (83%) | PE (CXR/USG):  34/42 (81%) |  | Mortality: 8 (19%)  Mechanical ventilation: 12 (29%) |
| Weerakoon et al, 2011, Sri Lanka[83]  Prospective | N=166, all SD | HAI, IgM/IgG, ELISA, RT-PCR  SD: 166 |  | Ascites: 29 (17%) | PE: 41 (25%) |  | Mortality: 11 |
| Yousaf et al, 2011, Pakistan[87]  Prospective | N=158, hospitalized  Mean age: 29 (range 12-70) | Serologically - unknown specific method  DF: 103 (65%)  DHF: 49 (31%)  DSS: 6 (4%) |  | PE: 126 (80%)  - Day 1-3: 72 (46%)  - Day 7: 91 (58%)  - DF: 72/103 (70%)  - DHF: 48/49 (98%)  - DSS: 6/6 (100%)  Ascites: 37 (23%) |  |  |  |
| Gonzalez et al, 2008, Colombia[30]  Retrospective | N = 328, hospitalized (USG in 42, CXR in 17)  M: 163 (50%)  Median age: 25 yrs (range 0.25-80 yrs) | Clinical diagnosis  Diagnostic test (IgM**)** in 193 (59%) - of these, 82% were positive  DHF: 116 (35%) | Dyspnea: 16 (5%)  Tachypnea: 83 (25%)  Respiratory distress: 22 (7%) | Ascites: 32 /42 (80%)  Pleural effusion: 23/42 (57%) | Pleural effusion: 11/17 (79%)  Signs of fluid overload: 1 (7%) |  | Mortality: 1  ICU: 7 (2%) |
| Quiroz-Moreno et al, 2006, Mexico[65]  Prospective | N=132  M: 66 (47%)  Mean age: 38 (range 16-76) | IgM ELISA  DF: 21  DHF: 108  DSS: 3  Secondary: 49 (37%) |  | PE: 73/111 (66%)  - Bilateral: 16%  Ascites: 67 (60%) |  |  |  |
| Torres et al, 2004, Venezuela[80]  Prospective | N=112, hospitalized - all DHF/DSS  Adults > 15  Median age: 36 (range 15-92)  M: 64 (57%) | IgM + IgG by rapid qualitative immune-chromato-graphic test  DSS: 18 (16%) |  |  | PE: 14 (13%) |  | Mortality: 0 |
| Aziz et al, 2002, Bangladesh[9]  Cross-sectional | N=34  Mean age: 28±11 yrs, range 6-50  M: 30 (67%) | RT-PCR, ELISA for IgM + IgG |  | Ascites: 4 (11%)  Pleural effusion: 1 (3%) |  |  |  |
| ARDS = acute respiratory distress syndrome, CXR = chest X-ray, DEN = dengue, DF = dengue fever, DFWS = dengue fever with warning signs, DHF = dengue hemorrhagic fever DSS = dengue shock syndrome, HAI = hemagglutination–inhibition antibody titer, M = male, NSD = non-severe dengue , OFI = other febrile illness, PE = pleural effusion, PEI = pleural effusion index (100 ‧ max width of PE / max width of hemithorax), SD = severe dengue, USG = ultrasonography | | | | | | | |

|  | Number of studies reporting, n(%) | Mean frequency (%) | Mean frequency in patients with PE |
| --- | --- | --- | --- |
| Dengue characteristics |  |  |  |
| Serotype, DENV1 | 14 (16%) | 30% |  |
| Serotype, DENV2 | 14 (16%) | 12% |  |
| Secondary infection | 18 (21%) | 54% | 91% (n=1) |
| Clinical complication |  |  |  |
| Dyspnea | 15 (18%) | 23% | 52% (n=2) |
| Acute respiratory distress | 19 (22%) | 9% | 61% (n=2) |
| Hemoptysis | 7 (8%) | 11% | - |
| Ascites | 61 (72%) | 24% | 45% (n=7) |
| Mortality | 48 (56%) | 6% | 46% (n=3) |
| DENV: dengue virus, PE: pleural effusion | | | |

## Supplemental Table 4: Dengue characteristics and clinical complications

## Supplemental Table 5: Dengue virus serotypes and pleural effusion

| **Study** | **Population** | **DENV-1, n(%)** | **DENV-2, n(%)** | **DENV-3, n(%)** | **DENV-4, n(%)** | **PE, n(%)** |
| --- | --- | --- | --- | --- | --- | --- |
| Aziz et al[9] | n=34 | 0 | 4  (12%) | 17  (50%) | 1  (3%) | 1  (3%) |
| da Costa Faria et al[18] | n=94  PCR in 50 | 0 | 16  (32%) | 0 | 34  (68%) | 4  (4%) |
| Dhanoa et al[22] | n=262  PCR in 222 | 170 (77%) | 44  (20%) | 7  (3%) | 1  (0.5%) | 6  (2%) |
| Fried et al[26] | n=457 | 162 (35%) | 102 (22%) | 123  (27%) | 64  (14%) | 167  (37%) |
| Hu et al[88] | n =38 | 33  (87%) | 5  (13%) | 0 | 0 | 12  (32%) |
| Huang et al[36] | n=1300  PCR in 937 | 14  (2%) | 823 (88%) | 99  (11%) | 1  (0.1%) | 181  (14%) |
| Jain et al[39] | n=369  PCR in 17 | 0 | 13  (76%) | 0 | 4  (24%) | 180  (49%) |
| Kabra et al[42] | n=15 | 0 | 15 (100%) | 0 | 0 | 13  (87%) |
| Lee et al[47] | n=1253  PCR in 801 | 59  (7%) | 658 (82%) | 83  (10%) | 1  (0.1%) | 79  (6%) |
| Malavige et al[48] | n=133 | 90  (68%) | 9  (7%) | 0 | 16  (12%) | 12  (9%) |
| Srikiatchorn et al[76] | n=82  PCR in 80 | 27  (34%) | 10  (13%) | 7  (9%) | 36  (45%) | 26  (32%) |
| Tassniyom et al[79] | n=95 | 25  (26%) | 5  (5%) | 1  (1%) | 5  (5%) | N/A |
| Yacoub et al[86] | n=102  PCR in 65 | 52  (80%) | 10  (15%) | 0 | 3  (5%) | 25  (25%) |
| DENV: denugue virus; PCR: polymerase chain reaction, PE: pleural effusion | | | | | | |

## References

1. World Health Organization. Dengue haemorrhagic fever: Diagnosis, treatment, prevention and control, 2nd edition. WHO. 1997.

2. World Health Organization (WHO). DENGUE GUIDELINES FOR DIAGNOSIS, TREATMENT, PREVENTION AND CONTROL. WHO. 2009. doi:10.1176/pn.41.1.0029b

3. Acharyya A, Ghosh K, Bhattacharyya A, Ghosh M, Chakraborty S, Ghosh S, et al. The dengue fever and its complication: A scenario in a tertiary-level hospital of greater Kolkata. Ann Trop Med Public Heal. 2016;9: 92–96. doi:10.4103/1755-6783.177375

4. Agarwal N, Roy MP, Singh MK. Clinical and Biochemical Findings in Confirmed Pediatric Dengue Cases in Delhi. J Pediatr Infect Dis. 2018;13: 15–19. doi:10.1055/s-0037-1602844

5. Agarwal N, Jain P. Sonography in dengue fever: An adjunct to clinico-laboratory profile. Indian J Public Heal Res Dev. 2016;7: 299–303. doi:10.5958/0976-5506.2016.00238.2

6. Ahlawat R, Kalra T. Atypical manifestations of dengue fever in a recent dengue outbreak. Ann Trop Med Public Heal. 2017;10: 1448. doi:10.4103/ATMPH.ATMPH_18_17

7. Ahmed FU, Mahmood CB, Sharma J Das, Hoque SM, Zaman R, Hasan MS. Dengue and dengue haemorrhagic fever in children during the 2000 outbreak in Chittagong, Bangladesh. Dengue Bull. 2001;25: 33–39.

8. Asghar J, Farooq K. Radiological appearance and their significance in the management of dengue hemorrhagic fever. Pakistan J Med Heal Sci. 2011;5: 685–692.

9. Aziz MM, Hasan KN, Hasanat MA, Siddiqui MA, Salimullah M, Chowdhury AK, et al. Predominance of the DEN-3 genotype during the recent dengue outbreak in Bangladesh. Southeast Asian J Trop Med Public Health. 2002;33: 42–8.

10. Bajaj S, D RC. Study of Ultrasound Finding in Dengue Fever. J Evid Based Med Healthc. 2016;3: 4683–4687. doi:10.18410/jebmh/2016/986

11. Balasubramanian S, Janakiraman L, Shiv Kumar S, Muralinath S, Shivbalan S. A reappraisal of the criteria to diagnose plasma leakage in dengue hemorrhagic fever. Indian Pediatr. 2006;43: 334–339.

12. Bandyopadhyay D, Chattaraj S, Hajra A, Mukhopadhyay S, Ganesan V. A study on spectrum of hepatobiliary dysfunctions and pattern of liver involvement in dengue infection. J Clin Diagnostic Res. 2016;10: OC21–OC26. doi:10.7860/JCDR/2016/16946.7784

13. Bhoi SK, Naik S, Kumar S, Phadke RV, Kalita J, Misra UK. Cranial imaging findings in dengue virus infection. J Neurol Sci. 2014;342: 36–41. doi:10.1016/j.jns.2014.04.018

14. Cam B V., Tuan DT, Fonsmark L, Poulsen A, Tien NM, Tuan HM, et al. Randomized comparison of oxygen mask treatment vs. nasal continuous positive airway pressure in Dengue Shock Syndrome with acute respiratory failure. J Trop Pediatr. 2002;48: 335–339. doi:10.1093/tropej/48.6.335

15. Chacko B, Subramanian G. Clinical, laboratory and radiological parameters in children with dengue fever and predictive factors for dengue shock syndrome. J Trop Pediatr. 2008;54: 137–140. doi:10.1093/tropej/fmm084

16. Chai XT, Baharuddin KA, Wahab SFA, Rahman A, Isa RM, Siti-Azrin AH. Ultrasound findings of plasma leakage as imaging adjunct in clinical management of dengue fever without warning signs. Med J Malaysia. 2020;75: 635–641.

17. Chandak S, Kumar A. Can radiology play a role in early diagnosis of dengue fever? N Am J Med Sci. 2016;8: 100–105. doi:10.4103/1947-2714.177316

18. Da Costa Faria NR, Solorzano VEF, De Souza LJ, Nogueira RMR, Bruycker-Nogueira F De, Chouin-Carneiro T, et al. Analysis of clinical and laboratory alterations related to dengue case severity: Comparison between serotypes 2 and 4 in Brazil. Am J Trop Med Hyg. 2017;97: 137–145. doi:10.4269/ajtmh.16-0227

19. Dayananda Kumar KR, Halawar RS. Comparative study of ultrasound findings in seropositive pediatric and adult patients with dengue fever. Radiol Infect Dis. 2018;5: 59–62. doi:10.1016/j.jrid.2018.04.001

20. de Kruif MD, Setiati TE, Mairuhu ATA, Koraka P, Aberson HA, Spek CA, et al. Differential gene expression changes in children with severe dengue virus infections. PLoS Negl Trop Dis. 2008;2. doi:10.1371/journal.pntd.0000215

21. Deshwal R, Qureshi MI, Singh R. Clinical and Laboratory Profile of Dengue Fever. J Assoc Physicians India. 2015;63: 30–32.

22. Dhanoa A, Hassan SS, Ngim CF, Lau CF, Chan TS, Adnan NAA, et al. Impact of dengue virus (DENV) co-infection on clinical manifestations, disease severity and laboratory parameters. BMC Infect Dis. 2016;16. doi:10.1186/s12879-016-1731-8

23. Dhooria GS, Bhat D, Bains HS. Clinical profile and outcome in children of dengue hemorrhagic fever in north India. Iran J Pediatr. 2008;18: 222–228.

24. Djamiatun K, van der Ven AJAM, de Groot PG, Faradz SMH, Hapsari D, Dolmans WMV, et al. Severe dengue is associated with consumption of von Willebrand factor and its cleaving enzyme ADAMTS-13. PLoS Negl Trop Dis. 2012;6: 1–8. doi:10.1371/journal.pntd.0001628

25. Ejaz K, Khursheed M, Raza A. Pleural effusion in dengue: Karachi perspective. Saudi Med J. 2011;32: 46–49.

26. Fried JR, Gibbons R V., Kalayanarooj S, Thomas SJ, Srikiatkhachorn A, Yoon IK, et al. Serotype-specific differences in the risk of dengue hemorrhagic fever: An analysis of data collected in Bangkok, Thailand from 1994 to 2006. PLoS Negl Trop Dis. 2010;4: 1–6. doi:10.1371/journal.pntd.0000617

27. Giraldo Rios D, Sant’Anna CC, March MDFBP, Abreu TF, Ferreira S, Bomfim M, et al. Pleuropulmonary manifestations of dengue fever in children and adolescents. J Pediatr Infect Dis. 2010;5: 363–367. doi:10.3233/JPI-2010-0281

28. Godbole V, Rana H, Mehta K, Gosai F. Rising trend of cases of dengue fever admitted in a tertiary care hospital in Vadodara – A retrospective study. Apollo Med. 2014;11: 255–260. doi:10.1016/j.apme.2014.10.001

29. González D, Castro OE, Kourí G, Perez J, Martinez E, Vazquez S, et al. Classical dengue hemorrhagic fever resulting from two dengue infections spaced 20 years or more apart: Havana, Dengue 3 epidemic, 2001-2002. Int J Infect Dis. 2005;9: 280–285. doi:10.1016/j.ijid.2004.07.012

30. González AL, Martínez RA, Villar LÁ. Evolución clínica de pacientes hospitalizados por dengue en una institución de salud de Bucaramanga, Colombia. Biomédica. 2008;28: 531. doi:10.7705/biomedica.v28i4.58

31. Goyal V, Singh Gill G, Singh J, Pratap Singh G, Singh Y, Singh S, et al. Clinical spectrums of dengue fever in a tertiary care centre with particular references to atypical presentation in the 2011 outbreak at Bathinda, Punjab, India. Int J Pharm Pharm Sci. 2013;5: 363–367.

32. Gupta AK, Peshattiwar P, Romday R, Bhambani P. A Study of Clinical and Laboratory Profile of Dengue Fever cases in a Tertiary Care Teaching Hospital. Int J Curr Microbiol Appl Sci. 2016;5: 295–307. doi:10.20546/ijcmas.2016.512.032

33. Gupta S, Singh L, Tandon R. Study of Pulmonary Manifestations among Dengue Patients in Tertiary Care Hospital of North India. Int J Contemp Med Res [IJCMR]. 2020;7: 1–5. doi:10.21276/ijcmr.2020.7.5.11

34. Hayat DES, Sultan A, Adeel Z, Fatima S, Ali M, Kumar B. Spectrum and Frequency of Imaging Findings in Dengue Fever. Pakistan J Med Heal Sci. 2020;14: 1749–1752.

35. Hu T, Liu J, Guan W, Zhang L, Jiang S, Chen B, et al. CT findings of severe dengue fever in the chest and abdomen. Radiol Infect Dis. 2015;2: 77–80. doi:10.1016/j.jrid.2015.08.002

36. Huang WC, Lee IK, Chen YC, Tsai CY, Liu JW. Characteristics and predictors for gastrointestinal hemorrhage among adult patients with dengue virus infection: Emphasizing the impact of existing comorbid disease(s). PLoS One. 2018;13: 1–12. doi:10.1371/journal.pone.0192919

37. Islam QT, Basher A, Amin R. Dengue: A practical experience of medical professionals in hospital. J Med. 2012;13: 160–164. doi:10.3329/jom.v13i2.12751

38. Jain D, Rajput R, Pathak V, Mittal A, Jain P. Changing trends in clinical presentation and biochemical spectrum of dengue fever: An observation of a tertiary care centre. Arch Clin Infect Dis. 2017;12. doi:10.5812/archcid.62221

39. Jain S, Mittal A, Sharma SK, Upadhyay AD, Pandey RM, Sinha S, et al. Predictors of dengue-related mortality and disease severity in a tertiary care center in north India. Open Forum Infect Dis. 2017;4: 1–8. doi:10.1093/ofid/ofx056

40. Jain V, Khan A, Garg R, Chopra A, Gaur D, Kashyap VK, et al. Discriminating malaria and dengue fever in endemic areas: Clinical, biochemical and radiological criteria. Clin Epidemiol Glob Heal. 2020;8: 1204–1207. doi:10.1016/j.cegh.2020.04.014

41. Joshi R, Baid V. Profile of dengue patients admitted to a tertiary care hospital in Mumbai. Turk J Pediatr. 2011;53: 626–631.

42. Kabra SK, Verma IC, Arora NK, Jain Y, Kalra V. Dengue haemorrhagic fever in children in Delhi. Bull World Health Organ. 1992;70: 105–108.

43. Kalayanarooj S, Vaughn DW, Nimmannitya S, Green S, Suntayakorn S, Kunentrasai N, et al. Early clinical and laboratory indicators of acute dengue illness. J Infect Dis. 1997;176: 313–321. doi:10.1086/514047

44. Kasim YA, Anky Tri Rini KE, Sumarmo SP. Hyperventilation in children with dengue hemorrhagic fever (DHF). Paediatr Indones. 1991;31: 245–252. doi:10.14238/pi31.9-10.1991.245-56

45. Khurram M, Qayyum W, Umar M, Jawad M, Mumtaz S, Khaar HTB. Ultrasonographic pattern of plasma leak in dengue haemorrhagic fever. J Pak Med Assoc. 2016;66: 260–264.

46. Kuo HJ, Lee IK, Liu JW. Analyses of clinical and laboratory characteristics of dengue adults at their hospital presentations based on the World Health Organization clinical-phase framework: Emphasizing risk of severe dengue in the elderly. J Microbiol Immunol Infect. 2018;51: 740–748. doi:10.1016/j.jmii.2016.08.024

47. Lee IK, Liu JW, Chen YH, Chen YC, Tsai CY, Huang SY, et al. Development of a simple clinical risk score for early prediction of severe dengue in adult patients. PLoS One. 2016;11. doi:10.1371/journal.pone.0154772

48. Malavige GN, Wijewickrama A, Fernando S, Jeewandara C, Ginneliya A, Samarasekara S, et al. A preliminary study on efficacy of rupatadine for the treatment of acute dengue infection. Sci Rep. 2018;8: 1–14. doi:10.1038/s41598-018-22285-x

49. Manam G, Godavarthi RM, Baru R, Sunitha S, Duddu GS. Evaluation of Ultrasonographic Findings in Dengue Fever Cases during an Outbreak at a Tertiary Care Hospital of South India. Int J Contemp Med Surg Radiol. 2018;3: 106–110. doi:10.21276/ijcmsr.2018.3.2.26

50. Michels M, Sumardi U, de Mast Q, Jusuf H, Puspita M, Dewi IMW, et al. The Predictive Diagnostic Value of Serial Daily Bedside Ultrasonography for Severe Dengue in Indonesian Adults. PLoS Negl Trop Dis. 2013;7. doi:10.1371/journal.pntd.0002277

51. Mishra VN, Motiramani N. Clinical and laboratory profile of dengue fever. J Assoc Physicians India. 2016;64: 102.

52. Mohamed NA, El-Raoof EA, Ibraheem HA. Respiratory manifestations of dengue fever in Taiz-Yemen. Egypt J Chest Dis Tuberc. 2013;62: 319–323. doi:10.1016/j.ejcdt.2013.03.002

53. Motla M, Manaktala S, Gupta V, Aggarwal M, Bhoi SK, Aggarwal P, et al. Sonographic evidence of ascites, pleura-pericardial effusion and gallbladder wall edema for dengue fever. Prehosp Disaster Med. 2011;26: 335–341. doi:10.1017/S1049023X11006637

54. Nainggolan L, Wiguna C, Hasan I, Dewiasty E. Gallbladder Wall Thickening for Early Detection of Plasma Leakage in Dengue Infected Adult Patients. Acta Med Indones. 2018;50: 193–199.

55. Oliveira GA, MacHado RC, Horvat JV, Gomes LE, Guerra LR, Vandesteen L, et al. Transient reticular gallbladder wall thickening in severe Dengue fever: A reliable sign of plasma leakage. Pediatr Radiol. 2010;40: 720–724. doi:10.1007/s00247-009-1489-x

56. Parmar J, Mohan C, Prem Kumar G, Vora M. Ultrasound is not useful as a screening tool for dengue fever. Polish J Radiol. 2017;82: 693–700. doi:10.12659/PJR.902861

57. Parmar J, Vora M, Mohan C, Shah S, Mahajan H, Patel T. ”Honeycomb” pattern of gallbladder wall thickening – A forward step in early diagnosis of “Severe Dengue Fever.” Indian J Radiol Imaging. 2019;29: 14–18. doi:10.4103/ijri.IJRI_363_18

58. Pereira MS, Kudru CU, Nair S, Thunga G, Kunhikatta V, Guddattu V. Factors associated with severity of illness in patients with dengue fever in a tertiary care hospital in southern India. Asian J Pharm Clin Res. 2018;11: 272–276. doi:10.22159/ajpcr.2018.v11i3.23496

59. Phung N, Thi T, Pham K, Tran DT. Respiratory Distress Associated with Dengue Hemorrhagic Fever on Paediatric Patients : Learning from a Provincial Hospital in Southern Vietnam. Arch Pharm Pract. 2019;10: 92–97.

60. Pone SM, Hökerberg YHM, de Oliveira R de VC, Daumas RP, Pone TM, Pone MV da S, et al. Sinais clínicos e laboratoriais para o dengue com evolução grave em crianças hospitalizadas. J Pediatr (Rio J). 2016;92: 464–471. doi:10.1016/j.jped.2015.12.005

61. Pothapregada S, Kamalakannan B, Thulasingham M, Sampath S. Clinically profiling pediatric patients with dengue. J Glob Infect Dis. 2016;8: 115–120. doi:10.4103/0974-777X.188596

62. Pramuljo HS, Harun SR. Ultrasound findings in dengue haemorrhagic fever. Pediatr Radiol. 1991;21: 100–102. doi:10.1007/BF02015615

63. Prasad D, Kumar C, Jain A, Kumar R. Accuracy and applicability of the revised WHO classification (2009) of dengue in children seen at a tertiary healthcare facility in northern India. Infection. 2013;41: 775–782. doi:10.1007/s15010-013-0405-3

64. Premaratna R, Ragupathy A, Miththinda N, de Silva J. Predictors of duration and degree of third space fluid accumulation in adult patients with dengue. Int J Infect Dis. 2012;16: e98. doi:10.1016/j.ijid.2012.05.227

65. Quiroz-Moreno R, Méndez GF, Ovando-Rivera KM. Utilidad clínica del ultrasonido en la identificación de dengue hemorrágico. Rev Med Inst Mex Seguro Soc. 2006;44: 243–248.

66. Rathi M, Masand R, Purohit A. Study of Dengue Infection in Rural Rajasthan. J Evol Med Dent Sci. 2015;4: 6849–6859. doi:10.14260/jemds/2015/993

67. Rathore APS, Senanayake M, Athapathu AS, Gunasena S, Karunaratna I, Leong WY, et al. Serum chymase levels correlate with severe dengue warning signs and clinical fluid accumulation in hospitalized pediatric patients. Sci Rep. 2020;10: 1–11. doi:10.1038/s41598-020-68844-z

68. Rodrigues RS, Brum ALG, Paes MV, Póvoa TF, Basilio-de-Oliveira CA, Marchiori E, et al. Lung in dengue: Computed tomography findings. PLoS One. 2014;9. doi:10.1371/journal.pone.0096313

69. Sahana KS, Sujatha R. Clinical Profile of Dengue Among Children According to Revised WHO Classification: Analysis of a 2012 Outbreak from Southern India. Indian J Pediatr. 2015;82: 109–113. doi:10.1007/s12098-014-1523-3

70. Santhosh V, Patil P, Srinath M, Kumar A, Jain A, Archana M. Sonography in the diagnosis and assessment of dengue fever. J Clin Imaging Sci. 2014;4: 1–7. doi:10.4103/2156-7514.129260

71. Schmitz L, Prayag S, Varghese S, Jog S, Bhargav-Patil P, Yadav A, et al. Nonhematological organ dysfunction and positive fluid balance are important determinants of outcome in adults with severe dengue infection: A multicenter study from India. J Crit Care. 2011;26: 441–448. doi:10.1016/j.jcrc.2011.05.008

72. Setiawan MW, Samsi TK, Wulur H, Sugianto D, Pool TN. Dengue haemorrhagic fever: Ultrasound as an aid to predict the severity of the disease. Pediatr Radiol. 1998;28: 1–4. doi:10.1007/s002470050281

73. Shabbir M, Ameen F, Roshan N, Israr M. Nature and Clinical Course of Pleural Effusion in Dengue Fever. Int J Intern Emerg Med. 2018;1: 1006.

74. Shah I, Deshpande GC, Tardeja PN. Outbreak of dengue in Mumbai and predictive markers for dengue shock syndrome. J Trop Pediatr. 2004;50: 301–305. doi:10.1093/tropej/50.5.301

75. Soller B, Srikiatkachorn A, Zou F, Rothman AL, Yoon IK, Gibbons R V., et al. Preliminary evaluation of near infrared spectroscopy as a method to detect plasma leakage in children with dengue hemorrhagic fever. BMC Infect Dis. 2014;14: 1–6. doi:10.1186/1471-2334-14-396

76. Srikiatkhachorn A, Krautrachue A, Ratanaprakarn W, Wongtapradit L, Nithipanya N, Kalayanarooj S, et al. Natural History of Plasma Leakage in Dengue Hemorrhagic Fever. Pediatr Infect Dis J. 2007;26: 283–290. doi:10.1097/01.inf.0000258612.26743.10

77. Srinivasa S, Nawab T, Nair CC. Clinical profile and ultasonogaphic findings in children with dengue fever. Curr Pediatr Res. 2014;18: 87–90.

78. Kumar Sudarsi R, Gundam B, Abhishek A. A CLINICAL PROFILE OF DENGUE FEVER IN OSMANIA GENERAL HOSPITAL. J Evol Med Dent Sci. 2016;5: 5035–5040. doi:10.14260/jemds/2016/1143

79. Tassniyom S, Vasanawathana S, Dhiensiri T, Nisalak A, Chirawatkul A. Failure of carbazochrome sodium sulfonate (AC-17) to prevent dengue vascular permeability or shock: A randomized, controlled trial. J Pediatr. 1997;131: 525–528. doi:10.1016/S0022-3476(97)70055-6

80. Torres JR, Torres-Viera JM, García H, Silva JR, Baddour Y, Bajares A, et al. Prognostic factors of clinical outcome in non-paediatric patients with dengue haemorrhagic fever/dengue shock syndrome. Dengue Bull. 2004;28: 68–74.

81. Venkata Sai PM, Dev B, Krishnan R. Role of ultrasound in dengue fever. Br J Radiol. 2005;78: 416–418. doi:10.1259/bjr/54704044

82. Voraphani N, Theamboonlers A, Khongphatthanayothin A, Srisai C, Poovorawan Y. Increased level of hepatocyte growth factor in children with dengue virus infection. Ann Trop Paediatr. 2010;30: 213–218. doi:10.1179/146532810X12786388978607

83. Weerakoon KG, Kularatne SA, Edussuriya DH, Kodikara SK, Gunatilake LP, Pinto VG, et al. Histopathological diagnosis of myocarditis in a dengue outbreak in Sri Lanka, 2009. BMC Res Notes. 2011;4: 2–7. doi:10.1186/1756-0500-4-268

84. Wu KL, Changchien CS, Kuo CH, Chiu KW, Lu SN, Kuo CM, et al. Early abdominal sonographic findings in patients with dengue fever. J Clin Ultrasound. 2004;32: 386–388. doi:10.1002/jcu.20060

85. Yacoub S, Griffiths A, Hong Chau TT, Simmons CP, Wills B, Hien TT, et al. Cardiac function in Vietnamese patients with different dengue severity grades. Crit Care Med. 2012;40: 477–483. doi:10.1097/CCM.0b013e318232d966

86. Yacoub S, Trung TH, Lam PK, Thien VHN, Hai DHT, Phan TQ, et al. Cardio-haemodynamic assessment and venous lactate in severe dengue: Relationship with recurrent shock and respiratory distress. PLoS Negl Trop Dis. 2017;11: 1–14. doi:10.1371/journal.pntd.0005740

87. Yousaf KR, Atiq S, Sheikh QS, Nisar MS, Mansoor Z, Khalid S. Sonographic features of polyserositis as an adjunct to clinico - Pathological parameters in diagnosing and predicting the severity of dengue fever. Pakistan J Med Heal Sci. 2011;5: 184–189.

88. Hu T, Liu J, Guan W, Zhang L, Jiang S, Chen B, et al. CT findings of severe dengue fever in the chest and abdomen. Radiol Infect Dis. 2015;2: 77–80. doi:10.1016/j.jrid.2015.08.002

89. S Y, A G, T.T HC, C.P S, B W, T.T H, et al. Cardiac function in Vietnamese patients with different dengue severity grades. Crit Care Med. 2012;40: 477–483. doi:10.1097/CCM.0b013e318232d966.Cardiac
